# Transposable element dynamics drive rapid evolution of centromere architecture in the *Avena* genus

**DOI:** 10.1186/s13059-026-04127-6

**Published:** 2026-06-16

**Authors:** Carlotta Marie Wehrkamp, Matthias Heuberger, Thomas Wicker

**Affiliations:** Department of Plant and Microbial Biology, University of Zurich, Zurich, Switzerland

**Keywords:** *Avena*, Centromere evolution, Poaceae, Transposable elements

## Abstract

**Background:**

Recent advances in assemblies of nearly gap-free, high-quality genomes have enabled detailed analysis of centromeres in large and highly repetitive crop genomes. Here, we analyze the centromeres of hexaploid oat (*Avena sativa*) and its tetraploid (*Avena insularis*) and diploid relatives (*Avena longiglumis*, *Avena atlantica* and *Avena eriantha*).

**Results:**

Avena centromeres are largely composed of retrotransposons belonging to three families, *RLG_Ava*, *RLG_Cereba* and *RLG_Beth*. Comparative analysis of retrotransposon populations reveals striking differences in centromere composition and architecture among the A, C and D subgenome lineages. We identify distinct profiles of transposable element bursts for these lineages, including the emergence and loss of retrotransposon families and subfamilies. Furthermore, we identify multiple inversions within centromeric regions of *Avena sativa* and retrace species divergences and polyploidization events in the *Avena* genus. Despite their close evolutionary relationships, our data show that the studied species exhibit rapid and divergent evolution of centromere architectures, for example through the spread of novel satellite repeats or activity bursts of different retrotransposon families and subfamilies. Additionally, we find that *RLG_Ava* and *RLG_Cereba* retrotransposons have been coexisting and possibly competing for the centromeric “niche” since the emergence of the Poaceae.

**Conclusions:**

Our comparative analyses provide detailed insight into centromere evolution across the *Avena* genus and reveal that composition and architecture of centromeres can vary greatly even between closely related species and different ploidy levels. Our findings emphasize the need for extended analyses of large genome species to improve our understanding of centromere evolution.

**Supplementary Information:**

The online version contains supplementary material available at 10.1186/s13059-026-04127-6.

## Background

Centromeres are essential in the cell division of eukaryotes. They are the assembly site of the kinetochore to which microtubules bind during mitosis and meiosis and thus mediate chromosome segregation. In most species, centromeres are defined epigenetically, rather than through a specific sequence of DNA, by the presence of the centromere specific CENH3 histone variant, which substitutes two canonical H3 histones [1]. There are two main types of centromeres: holocentromeres, in which the centromere stretches along the whole chromosome or where the spindle fibers attach the multiple CENH3 clusters (cluster-like holocentromere) and monocentromeres (point or regional centromeres), in which a single CENH3 containing region defines the functional centromere [2–4].

The Poaceae (grasses) family comprises all major cereal crops, for example wheat, barley, oat, maize, rye and rice. The most common centromere architecture found within the grass family are “regional” or monocentromeres, i.e. centromeres that comprise a defined, small part of chromosomes and are usually a few Mb in size [5, 6]. First evidence of centromere specific repetitive sequences in cereals was brought up in 1996 by Jiang et al. [7]. They demonstrated the presence of pSau3A9 (isolated from *Sorghum bicolor*) in the centromeric regions of various grass chromosomes including oat. In the same year Aragón-Alcaide et al. showed that the CCS1 (cereal centromeric sequence, isolated from *Brachypodium*) family occurred in the centromeres of the Triticeae, maize and rice [8]. Both sequences were later shown to be part of a centromere specific retrotransposon belonging to the *Gypsy* superfamily [9, 10]. They are conserved in the centromeres of grasses [9] and have repeatedly been shown to localize in the functional centromere by fluorescence in-situ hybridization assays (FISH) and chromatin immunoprecipitation (ChIP) (e.g. rice and maize) [11–13]. Most centromere-specific retrotransposons in grasses belong to the CRM clade, an ancient group of LTR retrotransposons [14]. These are called CRM in maize, CRR in rice and *Cereba* in barley [10, 12, 13]. Jiang and colleagues conducted FISH experiments using the Oligo-CCS1 against hexaploid oat and showed that there was no signal in the C subgenome chromosomes [15]. Furthermore, sequences from *Avena* species extracted using CCS1 primers were described to represent fragments of *RLG_Cereba* retrotransposons [16].

In recent years, the tribe of the Triticeae, which includes some of the world’s most important crops such as wheat, barley and rye, has become a model for the study of large and highly repetitive genomes. The dominant centromere-specific transposable element (TE) family in Triticeae is *RLG_Cereba* [7, 17–19]*.* Curiously, centromeres of Triticeae also contain a second, less abundant transposable element (TE) family called *RLG_Quinta*. *RLG_Quinta* is a non-autonomous retrotransposon, i.e. a TE family which does not encode canonical retrotransposon proteins, and which likely evolved from *RLG_Cereba* elements through loss of most of its coding sequences [20].

It has long been speculated that centromere specificity of centromere-specific TEs is due to a unique protein domain that is fused to the C-terminus of the integrase enzyme (called CR-domain) [14, 21, 22]. Retrotransposons containing this domain were named the CHDCR group, which is a sub-clade of CRM elements [14].

Recently, Heuberger et al. [20] proposed that the centromere specificity of *RLG_Cereba* might be rooted in a conserved RARA[RK]-motive in the CR-domain directly interacting with the CENH3 histone variant. Additionally, the study proposed that the long terminal repeats (LTRs) of *RLG_Cereba* and its non-autonomous partner, *RLG_Quinta*, are involved in the phasing/placement of CENH3 containing nucleosomes. Interestingly, mechanisms to target centromeres seem to have evolved multiple times independently during evolution. For example, centromeres of the algae *Asterochloris mediterranea* are composed of *Gypsy* retrotransposons which encode a different type of chromodomain [23]. Additionally, the *Copia* element *Tal1* (*Transposon of Arabidopsis lyrata 1*, a copy of *ALE4*) showed targeted insertions into CENH3 chromatin in *Arabidopsis* [24], despite lacking a chromodomain.

Only a few Triticeae genomes have completely sequenced, gap-free centromeres (e.g. *T. monococcum* [5]). While centromeres of rye and several wheat genomes are partially sequenced [18, 25], the centromeres of barley remain largely unresolved due to the presence of highly repetitive tandem satellite repeats [26]. Many questions therefore remain unanswered about centromeres in the large-genome cereals. For example, hexaploid wheat contains an additional centromere-specific retrotransposon family called *RLG_Abia* [27]. However, these elements seem to have been silent during recent evolutionary times, as mostly fragments are found. Considering the rapid turnover of repetitive sequences in grasses (amplification of TEs and their continuous removal through deletions; [28]), this indicates that *RLG_Abia* elements became silent only relatively recently and suggests that different TE families may populate cereal centromeres at different times.

Recently, a highly contiguous genome of the hexaploid oat *Avena sativa* (ACD) was made available (*Avena sativa*—OT3098 v2, [29]). With a size of over 10 Gb, it is among the largest genomes sequenced so far. This was also the first assembly in which centromeres were assembled nearly gap free. Centromeres of the A and D subgenome were shown to consist mainly of LTR retrotransposons, while C subgenome centromeres also contained highly abundant tandem repeats [30–32]. Oat (*A. sativa*) is a close cereal relative to the Triticeae tribe which contains wheat, barley and rye. The Aveneae and Triticeae lineages diverged approximately 28 million years ago [33] while the Triticeae themselves radiated approximately 8–12 million years ago [33–35]. The genus *Avena* includes diploid, tetraploid and hexaploid species. *A. sativa* is an agronomically important allohexaploid crop (genome size = 10.78 Gb) comprising three large and highly repetitive A, C and D subgenomes.

The phylogeny of hexaploid oat has long been a topic of discussion. Different *Avena* species have been brought up as potential donors of the genomes found in hexaploid oats and especially the origin of the D genome has been the focus of recent studies. The A and D genome are thought to be more closely related to each other than to the C genome [36, 37]. In addition, it has been suggested that the D genome of hexaploid oats is derived from the AC tetraploids. And further, based on genome wide analysis and phylogenetic studies, it was proposed that these tetraploids carry a D genome instead of an A genome [38–41]. The tetraploid *Avena insularis* (CD, formerly AC) is presumed to be the progenitor of hexaploid oats with evidence being provided for example by chromosome morphology similarity [42, 43] through genetic diversity studies [44], high-density genotyping-by-sequencing markers [38] and recently by cytogenetic analysis using FISH [41].

On the other hand, a phylogenetic study from 2018 based on the *Pkg1* (nuclear plastid 3-phosphoglycerate kinase) gene, suggest that the tetraploids *Avena murphyi* or *Avena marrocana* might be a closer relative to the C and D genomes found in hexaploid oats [45], however the consensus seems to emerge that *A. insularis* is the probable CD genome donor. A high-quality reference genome for *A. insularis* was recently presented by Kamal et al. [31].

The origin of the A genome of hexaploid oats has been studied extensively, however no particular *Avena* species has been widely agreed on as a putative donor yet. Multiple studies analyzed among others the diploid *Avena longiglumis* (A_l_) [31, 35, 46–48] as a possible progenitor of the A genome in hexaploid oat. For example, in a recent study by Avni et al. presenting annotated assemblies of 33 wild and domesticated oat lines and highlighting structural variation amongst them, the authors refer to *A. longiglumis* as the closest extant relative of the diploid ancestor of hexaploid oats’ A subgenome [49]. Other studies highlighted that *Avena atlantica* (A_s_) is closely related to the A genome in hexaploid oat and discussed both of the above-mentioned diploids as putative diploid ancestors [33, 37, 45, 46]. Reference genomes were made available recently for *A. longiglumis* [31] and *A. atlantica* [50]. The comprehensive analysis of Zhang and colleagues indicates that the A_l_ lineage gave rise to the hexaploid ancestor of *A. sativa* [51]. In the following we use both, *A. longiglumis* and *A. atlantica,* for the analysis of centromeres of the *Avena* genus.

Using the above-mentioned genomes from the *Avena* genus, the goal of this study was to characterize the centromeric architecture in this genus, with particular focus on CRM TEs. We found that *Avena* centromeres in the A and D subgenomes are dominated by two putatively autonomous CRM retrotransposon families, *RLG_Cereba* and *RLG_Ava*, which seem to compete for the centromeric ”niche“ in different *Avena* species. In addition, we identified a putatively non-autonomous partner family to *RLG_Ava*, called *RLG_Beth*. We use the *RLG_Ava* and *RLG_Cereba* families to investigate the evolutionary history of multiple *Avena* genomes, with a focus on previously proposed progenitors of hexaploid oat. Finally, we explored the evolutionary origins of *RLG*_*Ava* and *RLG_Cereba* elements in the Poaceae and demonstrated their coexistence for millions of years.

## Results

### Two autonomous and one non-autonomous retrotransposon family are abundant in the centromeres of *A. sativa*

**Fig. 1:**
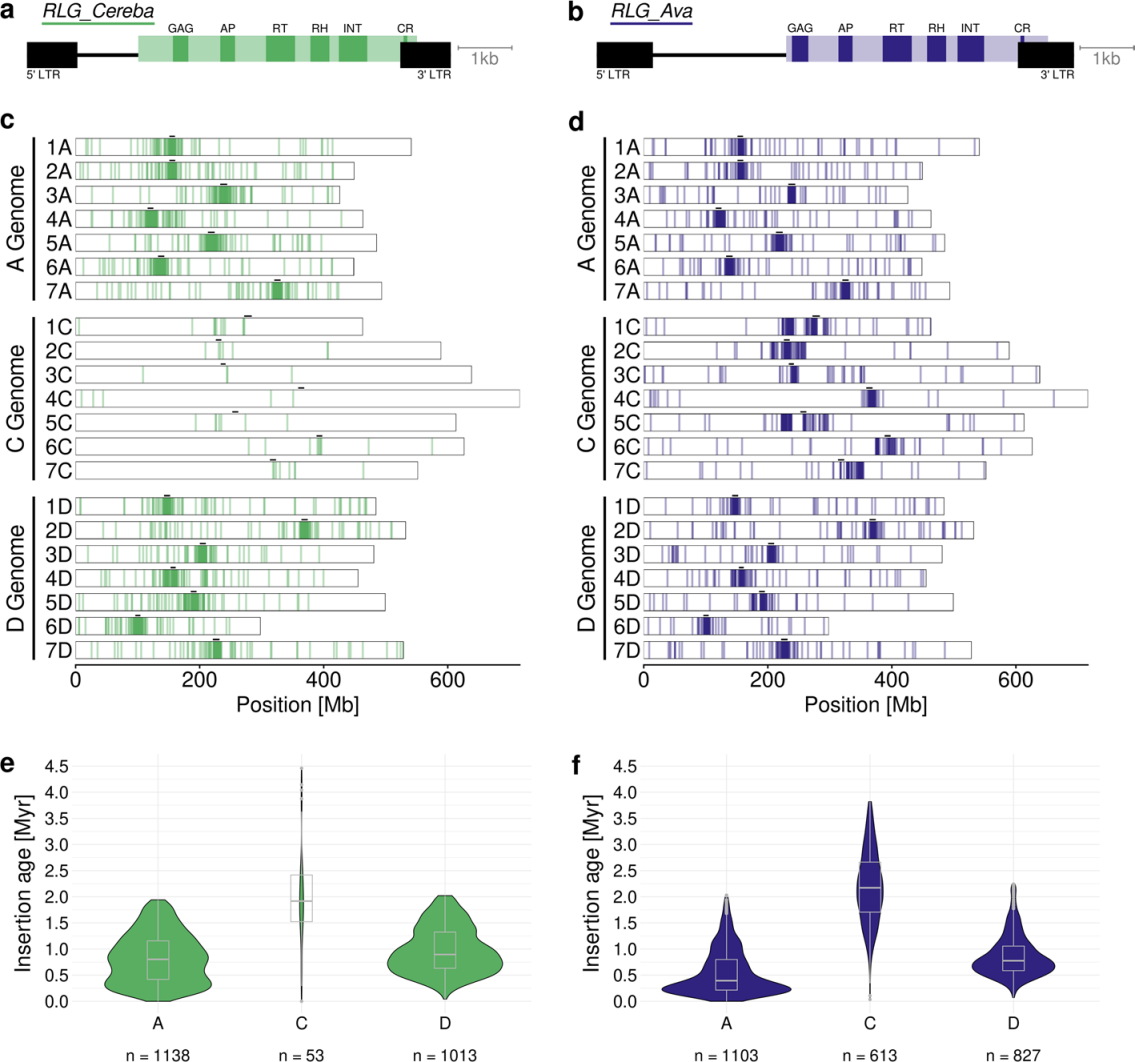
Distribution of centromere specific retrotransposons in *A. sativa* OT3098. Sequence organization of *RLG_Cereba* **a** and *RLG_Ava* elements **b** AP: aspartate protease, RT: reverse transcriptase, RH: RNase H, INT: integrase, CR: Chromodomain.** c** Chromosomal distribution of full-length *RLG_Cereba* retrotransposons. Inferred centromere positions are indicated by black bars. Note that the C subgenome contains only very low numbers of *RLG_Cereba* elements. **d** Chromosomal distribution of *RLG_Ava* retrotransposons*.* Inferred centromere positions are indicated by black bars. **e** Insertion age (in Myr) per subgenome of *RLG_Cereba* elements in *A. sativa* OT3098. **f** Insertion age (in Myr) per subgenome of *RLG_Ava* elements in *A. sativa* OT3098

We used an available CENH3 ChIP-seq dataset from *A. sativa* cv. Starter to infer centromere positions of *A. sativa* OT3098. We acknowledge that the inferred positions are estimations, as the dataset originated from a different accession (Additional file 1: Fig. S1, Tab. S1). However, this ChIP-seq dataset from *A. sativa* cv. Starter was recently also used to infer centromere positions in *A. sativa* cv. Sang [52]. Through EDTA annotation of the centromeric regions, we identified three main retrotransposon families (*RLG_Ava*, *RLG_Cereba*, *RLG_Beth*). *RLG_Cereba* from *A. sativa* shows an average sequence identity of ~ 76.5% across its entire length with the consensus sequence of *RLG_Cereba* from *Triticum monococcum*. *RLG_Ava* is similar to the previously described centromere-specific *RLG_Abia* retrotransposons from wheat [27]. Since *RLG_Ava* and *RLG_Abia* are only ~ 67% identical in their coding region and show little conservation in the LTRs, *RLG_Ava* was designated a new family (examples for comparisons in Additional file 1: Fig. S2 and Tab. S2). Both are putatively autonomous families, meaning they encode all proteins necessary for transposition and belong to the previously described CRM clade [14]. In addition, both families feature a CR domain with a RARA[RK]-motive located in the beginning of the 3’ LTR (Fig. 1a and b, see below) which places them in the CHDCR group of CRM elements [14].

When comparing consensus sequences of *RLG_Cereba* and *RLG_Ava*, we found the conservation to be too low to reliably align them on DNA level. Their predicted proteins show a similarity of 65.2% to 68.5%, indicating that these families represent distinct and ancient evolutionary lineages (see also phylogenetic analysis of CRM elements in Poaceae below). Additionally, we identified multiple variants of *RLG_Ava* elements which differ strongly in sequences of short tandem repeat clusters in their LTRs (example in Additional file 1: Fig. S3). Interestingly, we identified copies of the *RLG_Cereba* family almost exclusively in the A and the D subgenomes (Fig. 1c). For our analysis of TE populations, we defined full-length copies as those identified by the TEpop pipeline ([53], see “Methods”), meaning the respective TE consensus DNA sequence aligns over at least 99% of its length with an individual copy without major deletions or insertions. Full-length copies represent approximately 30% of the total sequence of a respective TE family, while the rest are fragmented through deletions or insertions of other TEs (Additional file 1: Fig. S4). We found 1138 and 1013 *RLG_Cereba* full-length copies in the A and D subgenome, respectively, while in the C subgenome we identified only 53 copies (Fig. 1e). In contrast, we found high numbers of *RLG_Ava* elements in all oat subgenomes, with 1103 copies in A, 613 in C and 827 copies in the D subgenome (Fig. 1d and f; Additional file 1: Fig. S5).

For all full-length copies, we estimated their insertion age based on divergence of their LTRs [54]. To ensure that insertion age estimates from different families are comparable, we first examined substitution rates in young copies. Substitution rates could differ, for example, due to differing error rates of the reverse transcriptases or from differing methylation rates between families. Our analysis indicated that substitution rates in different families and species are very similar (see “Methods”). We also found no evidence that TE families are differentially removed between species (which could otherwise also influence insertion age distributions, see “Methods”). Average insertion age of *RLG_Cereba* elements was estimated to be approximately 0.82 Myr in the A genome and ~ 0.98 Myr in the D genome, while the 53 copies in the C genomes were on average ~ 2 Myr old. Results were similar for *RLG_Ava* elements: while the A and D subgenome comprised elements with average ages of around ~ 0.55 and ~ 0.87 Myr, respectively, copies from the C subgenome were much older, with an average insertion age of ~ 2.2 Myr (Fig. 1e).

Of the third retrotransposon family (*RLG_Beth*), we found 1054, 171 and 930 full-length copies on the A, C and D subgenome, respectively. *RLG_Beth* copies also localize in the centromeric region of the chromosomes (Additional file 1: Fig. S6). *RLG_Beth* encodes a large protein of unknown function but does not encode the proteins required for transposition. We propose that *RLG_Beth* is a non-autonomous partner family to *RLG_Ava* and relies on the autonomous partner for its replication. Indeed, *RLG_Beth* and *RLG_Ava* show sequence conservation in their LTRs, primer binding sites and poly-purine tracts and a putative TATA-box (Additional file 1: Fig. S7). Furthermore, overall copy numbers and insertion ages of *RLG_Beth* elements are similar to those of *RLG_Ava* elements across *A. sativa* A and D subgenomes (Additional file 1: Fig. S5 and S6), indicating that they were active at the same time and at similar levels: copies in the C subgenome were estimated to be ~ 2.4 Myr old on average, while in the A and D subgenome the copies showed a lower insertion age of ~ 0.62 Myr and ~ 0.92 Myr respectively, very similar to those of *RLG_Ava* elements. These characteristics were previously described as criteria to define autonomous/non-autonomous pairs [19, 23, 53], we propose *RLG_Beth* to be a non-autonomous partner family to *RLG_Ava*, relying on the autonomous partner for its replication.

### Centromere-specific TEs indicate the positions of functional centromeres

We analyzed the positions of the most recent insertions of *RLG_Cereba*, because it was shown in wheat that the youngest *RLG_Cereba* copies were found inside or close to the functional centromere [5]. In oat, we found that most of the recently inserted *RLG_Cereba* elements, i.e. elements younger than 1 Myr, are highly enriched in the centromeres in subgenomes A and D (Additional file 1: Fig. S1, S8, Fig. S9). Likewise, *RLG_Ava* retrotransposons with insertion ages younger than 1 Myr were consistently enriched in inferred centromeres of subgenomes A and D (Additional file 1: Fig. S9). In fact, insertion sites of the youngest copies for both *RLG_Cereba* and *RLG_Ava* perfectly coincided with the inferred centromere positions in all chromosomes of the A and D subgenomes (Additional file 1: Fig. S1, S8 and S10). We thus concluded that young insertion of both *RLG_Cereba* and *RLG_Ava* are highly reliable markers for the centromeric regions in these two subgenomes. Indeed, between 68.7% and 86.6% of the CRM insertions with insertion ages at or below the 20th percentile in subgenomes A and D, i.e. the youngest insertions, are located within the centromeric region inferred through CENH3 ChIP-seq mapping (Additional file 1: Fig. S9c). This is in line with findings of previous studies that showed that insertions ages and distributions of centromere-specific TEs can be used to infer current and past centromere positions [5, 55].

However, the 613 *RLG_Ava* copies from the C subgenome were much older, with an average insertion age of ~ 2.2 Myr, while *RLG_Cereba* retrotransposons are virtually absent from the C genome (Fig. 1c). We therefore wanted to study whether these older elements also mark centromere positions. With the exception of elements localized in the previously described translocations on 1 C and 4 C (e.g., [31, 36, 56–58]), we found the youngest elements (in this case younger than 2 Myr) towards the peri-centromeric regions of the C subgenome with the exception of 7 C (Additional file 1: Fig. S1, S10). However, only up to 42.1% of CRM insertions with insertion ages at or below the 20th percentile in subgenome C are localized within the identified centromeres, thus, the CRMs cannot be regarded as reliable markers for the peri-centromeric region in this subgenome (Additional file 1: Fig. S9c). In summary, the CRM TEs *RLG_Cereba* and *RLG_Ava* in the A and D subgenomes are clearly younger than those in the C subgenome (Fig. 1e and f). Consequently, insertion of the youngest TE copies coincided well with the inferred positions of the centromeres in the A and D subgenomes. In contrast, *RLG_Ava* in the C subgenome seem to have been largely “silent” for a long time, suggesting that different dynamics might influence positions of functional centromeres in the C subgenome.

### C subgenome centromeres and chromosomes have a large fraction of tandem repeats

**Fig. 2:**
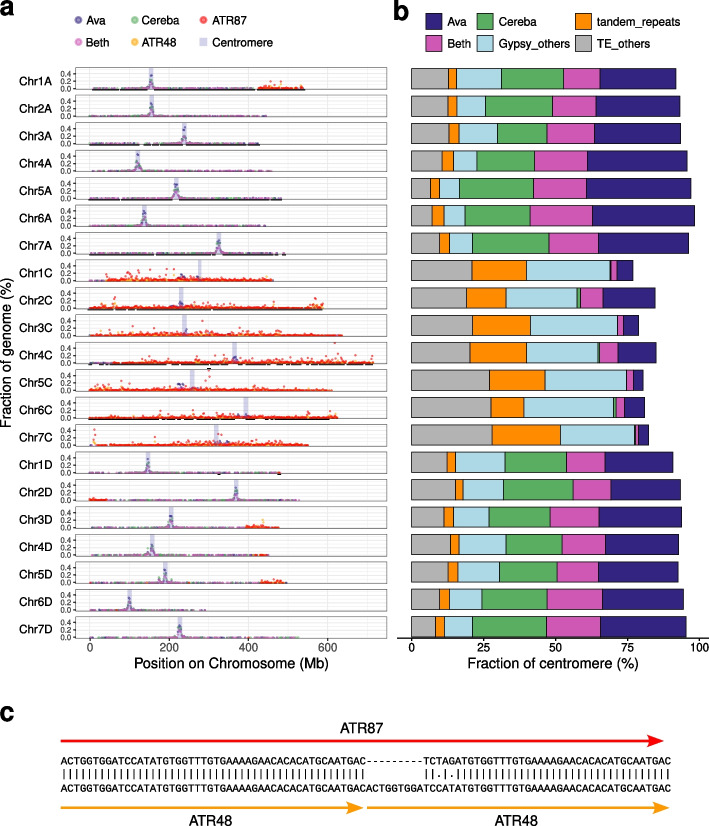
Repeat content of *Avena sativa* centromeres. **a** Fractions of the genome annotated as either *RLG_Ava*, *RLG_Beth*, *RLG_Cereba*, *ATR48* or *ATR87*, in 2 Mb bins. Note that *RLG_Ava*, *RLG_Beth* and *RLG_Cereba* are predominantly found in the centromeres, which are shaded in light blue. *ATR48* and *ATR87* are found throughout the chromosomes of the C subgenome as well as in specific chromosome segments in the A and D subgenomes, that represent translocations from the C to the other subgenomes. **b** Fractions of centromeres that are annotated as repeats. The A and D subgenome centromeres, mainly consist of *RLG_Ava*, *RLG_Beth* and *RLG_Cereba* TEs, whereas other repeats such as other TEs and tandem repeats are more prevalent in the C subgenome centromeres. **c** Sequence comparison of ATR87 and two repeat units of ATR47 showing that ATR87 is a higher order repeat derived from ATR47

To study the overall composition of *Avena* centromeres we annotated them with EDTA for TEs [59] and TRF for tandem repeats [60]. The A and D subgenome centromeres have low tandem repeat contents of 3.6% and 3.2%, respectively. In contrast, the C subgenome has a more than 6-fold higher tandem repeat content (19.6%), while ~ 60% are annotated as TEs (Fig. 2). A majority of the tandem repeat arrays found in the C genome centromeres consist of two repeats, one with a period size of 46–51 (majority 48), and one with a period size of mainly 87. These two tandem repeats were previously described as the centromeric *Cen48* and *Cen87* [30]. Although these two tandem repeats are present in the centromeres of the C subgenome, we also found them to be highly abundant along the entire chromosome arms of the C subgenome, as well as in known translocations of C subgenome origin into the A and D subgenomes [30] (Fig. 2). Considering that Cen48 and Cen87 are found across the entire C genome, we propose to change their names to ATR48 and ATR87 (Avena tandem repeat), to reflect that they are not specifically enriched in any genomic compartment. Additionally, ATR87 seems to be a higher order repeat derived from two units of ATR48 with an internal deletion (Fig. 2c). In summary, these results indicate that the C genome as a whole has experienced a massive amplification of tandem repeats after it diverged from the common ancestor genome. We also did not identify any other tandem repeats exclusive to centromeres in any of the three subgenomes.

Centromeres in all three subgenomes have a high TE content (A = 92.5%, C = 62.5%, D = 91.6%). Most TEs are of the *Gypsy* superfamily (A = 82.8%, C = 40.0%, D = 81.9%, of total centromeric DNA), of which *RLG_Ava* (A = 32.8%, D = 30.0%) and *RLG_Cereba* (A = 23.1%, D = 24.1%) are the most abundant in the A and D subgenomes. Together, tandem repeats and TEs account for 96.1% (A), 82.3% (C), and 91.6% (D) of the centromeres in *A. sativa,* meaning that the centromeric DNA is highly repetitive (Fig. 2). In summary, these analyses show that the A and D subgenomes have almost exclusively TE based centromere architectures, whereas the C subgenome differs in that it contains a considerable fraction of tandem repeats that are part of the centromere.

### Centromere specific TE insertion patterns suggest inversions in *A. sativa* centromeric regions

**Fig. 3:**
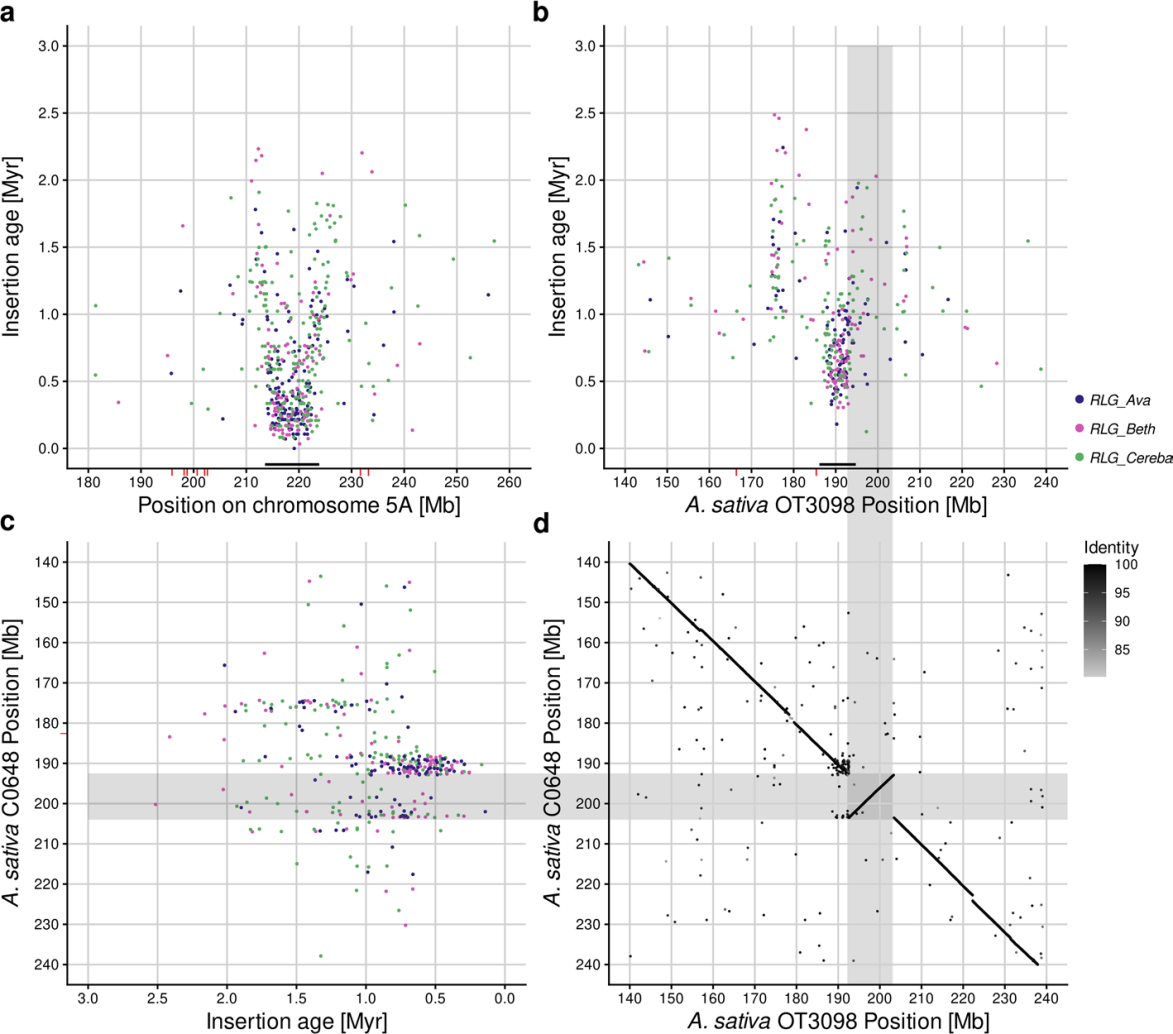
Estimated insertion ages of *RLG_Ava*, *RLG_Beth* and *RLG_Cereba* across the (peri-) centromeric region in chromosome 5 A (**a**) and 5D (**b**). Every point indicates one full-length element, colors indicate the family. Assembly gaps are shown by red marks on the x-axis. The black bars on bottom indicate the centromere position. Notice the continuous pattern of older insertions of these CRM TEs located towards the borders of the centromere in chromosome 5 A in comparison to chromosome 5D. Similarly, the distribution of CRMs is shown in **c** for *A. sativa* C0648. **d** Dot plot comparison of the (peri-) centromeric region of Chromosome 5D in *A. sativa* OT3098 and *A. sativa* C0468. Assembly gaps are indicated by red marks on the y and x-axis of the insertion age distribution plots for *A. sativa* C0468 and *A. sativa* OT3098 respectively. Gray boxes indicate the inversion. Note the distinct spatio-temporal cluster of CRM TE insertions at ~ 204 Mb in *A. sativa* C0648 which is not present in *A. sativa* OT3098

Due to insertion of CRM TEs into the functional centromere, i.e. in the vicinity of CENH3 marks, the youngest insertions are found within the functional centromere which in turn ‘pushes’ older copies outwards away from the centromere. This passive movement of elements in the past ~ 2 Myr can be seen for example in chromosome 5 A when investigating the distribution patterns of *RLG_Ava*, *RLG_Cereba* and *RLG_Beth* (Fig. 3a). Previous studies have shown that the distribution and insertion ages of LTR retrotransposons can be used to identify movements of centromeres over time (e.g. [5, 55]). On chromosome 5D, this pattern of passive outward movement of older copies is disrupted and distinct groups of CRM TEs can be observed, hereafter referred to as spatio-temporal clusters. These clusters could be indicative of inversion events in the (peri-) centromeric region of chromosome 5D. This proposed inversion possibly led to the discontinuity of the distribution pattern of CRM TE insertions and thus the establishment of the spatio-temporal cluster at ~ 175 Mb in *A. sativa* OT0398 (Fig. 3b). Similar distribution patterns can be seen in e.g., chromosomes 4D (Additional file 1: Fig. S1, S11). In chromosome 5 C we also identified spatio-temporal clusters around the centromere, but due to the high content of tandem repeats as well as inactivity of CRMs within the last ~ 1 Myr in the C subgenome, the distribution of the CRMs is much more spread out in comparison to chromosome 5 A (Additional file 1: Fig. S1). We also found similar patterns in other chromosomes of the C subgenome (Additional file 1: Fig. S1). We want to point out that this is indirect evidence suggesting putative inversions which need further investigation, ideally by comparison with assemblies of oat genotypes which do not carry this proposed inversion. However, we were indeed able to show that distributions and insertions ages of CRMs reflect inversions in the centromeric region in *Avena*: When comparing the distribution of insertion ages of CRMs in *A. sativa* OT3098 (Fig. 3b) and *A. sativa* C0648 [51] (Fig. 3c) on chromosome 5, we found a spatio-temporal cluster in C0648 at ~ 204 Mb which is absent in OT3098, indicating an inversion event in *A. sativa* C0648. Indeed, this cluster is located on the border of an Inversion found when comparing the (peri-) centromeric regions of the two genomes (Fig. 3d). Further, the chromosome 4D of *A. sterilis* [32] shows an inversion which is not present in the *A. sativa* OT3098 genome, and which produces a similar distinct spatio-temporal cluster of CRM copies as seen for the proposed inversion in 5D (Additional file 1: Fig. S11).

### Analysis of *RLG_Ava *and *RLG_Cereba *sheds light on *Avena* subgenome phylogeny

To study TE and centromere dynamics in the *Avena* genus, we expanded our analysis and included genome assemblies from two A-genome diploids, *A. longiglumis* and *A. atlantica* which carry the A_l_ and A_s_ genomes, respectively (e.g., [47, 61, 62]). Additionally, we included the genome of *A. insularis* (the presumed CD tetraploid progenitor of hexaploid oats) and *A. eriantha*, a diploid C genome relative carrying the C_p_ genome. We once more utilized the TE population analysis pipeline [53] and analyzed the *RLG_Ava* and *RLG_Cereba* families separately.

**Fig. 4:**
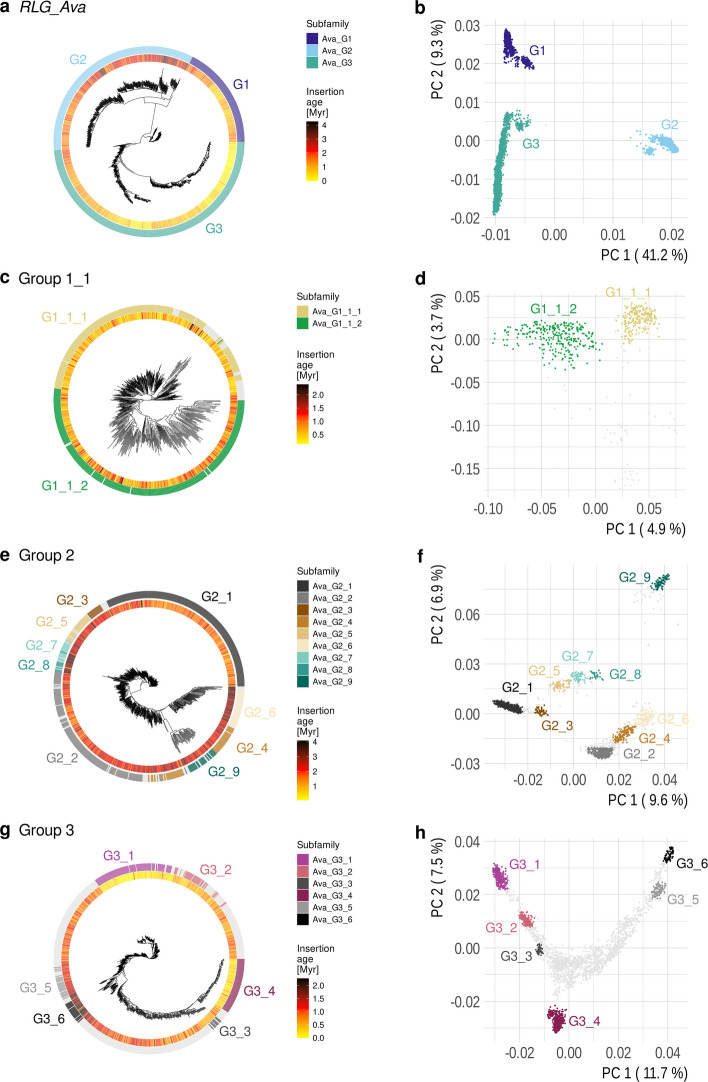
*RLG_Ava* elements from *A. sativa*, *A. longiglumis*, *A. atlantica*, *A. eriantha* and *A. insularis.* **a** Phylogenetic tree of 1250 randomly chosen copies of *RLG_Ava* elements with the main three groups (G1-G3) marked in shades of blue. Estimated insertion age is shown in the inner circle. Colors in the second circle indicate the selected subfamily. **b** The corresponding PCA from which subfamilies were selected, the colors indicate the subfamilies. Non-selected elements are shown in light grey. The same applies for a. **c** Phylogenetic tree of *RLG_Ava* randomly chosen 523 elements from group G1_1 (subset of G1, see Additional file 1: Fig. S12 for details). **d** The corresponding PCA for G1_1. **e** Phylogenetic tree of 750 randomly chosen *RLG_Ava* elements from group G2 with corresponding PCA in (**f**). **g** Phylogenetic tree of 1000 randomly chosen *RLG_Ava* elements from group G3 with corresponding PCA in (**h**)

**Fig. 5:**
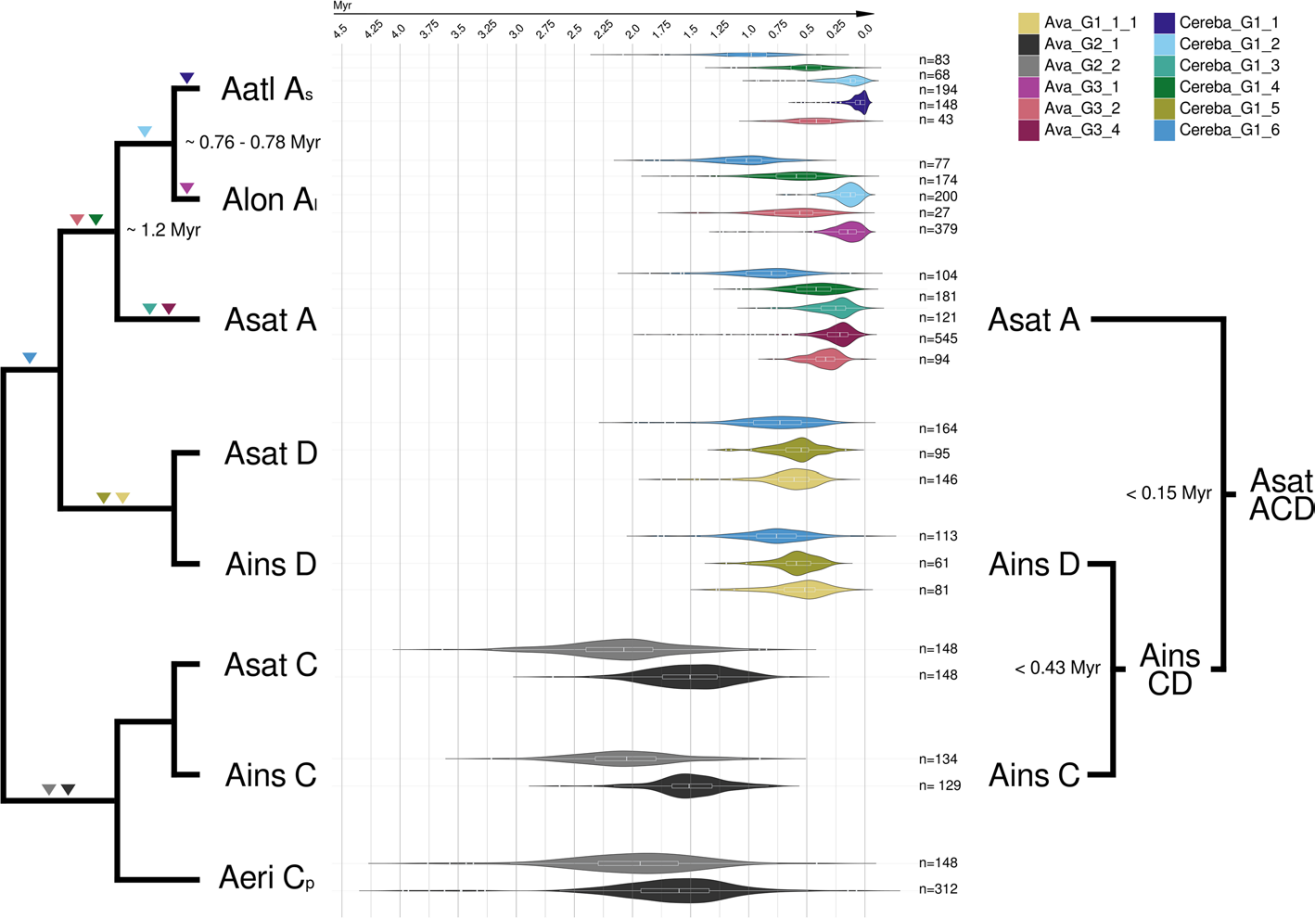
Graphic of the genetic relationship of different *Avena* species. The graphic displays the species *A. sativa* (ACD), *A. insularis* (CD), *A. longiglumis* (A_l_), *A. atlantica* (A_s_) and *A. eriantha* (C_p_). The triangles indicate the presence of a subfamily. The box plots highlight the time of activity bursts of the respective subfamily in the different genomes, the violin plot indicates the density. The names of the subfamilies were shortened

For *RLG_Ava*, our analysis revealed the identified 6,183 full-length copies to cluster into three distinct groups in the PCA based on SNPs (see “Methods” on TE population analysis), reflecting the three subgenomes (*RLG_Ava_G1* through *G3*; Additional file 1: Fig. S12). The three groups were divided up for a more detailed analysis (Additional file 1: Fig. S12) which led to the identification of 20 subfamilies in total, of which six provided distinct insight into the divergence and polyploidization history of the *Avena* (sub-) genomes, i.e. by being either common to a group of subgenomes or unique for one (Figs. 4 and 5). For *RLG_Cereba*, from a total of 5,165 full-length elements, we selected a subset which comprises the younger copies, as the subfamilies identified from using this subset were more diagnostic for the different genomes (Additional file 1: Fig. S13). From this subset of 3,040 full-length copies (*Cereba_G1*), we defined six subfamilies which provided insight into the *Avena* subgenome divergence and polyploidization (Figs. 4 and 5).

Particularly informative was subfamily *RLG_Cereba_G1_6*, which was found in all A and D genomes, indicating that it was present already in a common ancestor of the A and D genomes. The A/D genome specific presence of the subfamily is in line with previous findings that the A and D are more closely related to each other than to the C genome [37, 39]. Our analysis indicates that *RLG_Cereba_G1_6* had independent activity bursts in the A and D genome lineages between ~ 0.5 and ~ 1.2 Myr ago (Fig. 5). Here, we defined the duration of a TE activity burst as the time span that covers the inter quartile range (IQR, 25th to 75th percentile) of the insertion ages of a given subfamily. We know that the activity bursts in the A and D genome lineages must have been independent, as they overlap in time with activity bursts of subfamilies *RLG_Cereba_G1_5* and *RLG_Ava_G1_1_1* which are only found in the D genomes (Fig. 5). Therefore, the A and D genome lineages must have diverged before the *RLG_Cereba_G1_6* activity burst, at least ~ 1.2 Myr ago. For the D genomes of *A. sativa* and *A. insularis*, TE activity profiles of the three studied subfamilies look very similar (Fig. 5). This was expected, since *A. insularis* is the proposed progenitor of the hexaploid oats.

Interestingly, TE activity profiles in the three A genomes differed strongly and therefore provided information about their evolutionary history. For subfamily *RLG_Cereba_G1_4*, we identified independent bursts between the A genomes. In the *A. sativa* the beginning of the burst was estimated to ~ 0.59 Myr ago while in *A. atlantica* it started ~ 0.64 and in *A. longiglumis* ~ 0.76 Myr ago. Again, we conclude this must have been independent bursts because they overlap in time with activity bursts of subfamilies *RLG_Ava_G3_4* and *RLG_Cereba_G1_3* (Fig. 5) which are found only in *A. sativa*. This clearly distinguishes the *A. sativa* A subgenome from *A. longiglumis* and *A. atlantica*, taken together with the later onset of the burst of *RLG_Cereba_G1_6* in *A. sativa* this indicates that the *A. sativa* A subgenome diverged from the other two ~ 1.2 Myr ago.

In addition, we identified multiple subfamilies of *RLG_Cereba* and *RLG_Ava* that distinguish *A. longiglumis* and *A. atlantica.* The subfamily *RLG_Cereba_G1_1* which had an activity burst in the past 100,000 years is only found in *A. atlantica*, while subfamily *RLG_Ava_G3_1* is only found in *A. longiglumis.* Furthermore, subfamily *RLG_Ava_G3_2* had activity bursts at different times. While in the *A. longiglumis* the burst began ~ 0.78 Myr ago, in *A. atlantica* it started later at ~ 0.56 Myr (Fig. 5).

Taken together, the TE activity profiles indicated that *A. longiglumis* and *A. atlantica* must have diverged at least ~ 0.76 to 0.78 Myr, based on the independent burst of *RLG_Cereba_G1_4* and a later onset of the activity burst of *RLG_Ava_G3_2* in *A. atlantica*.

Further, we identified two large subfamilies (*RLG_Ava_G2_1* and *RLG_Ava_G2_2*) that were only present on the C genomes (Fig. 5). Consistent with results described above, the *RLG_Ava* subfamilies were active much earlier than in the A and D subgenomes and seem to be silent since at least 1 Myr (Fig. 5).

The activity profiles are also informative with regard to polyploidization events: the two subfamilies *RLG_Cereba_G1_5* and *RLG_Ava_G1_1_1* which are specific for the D genomes had activity bursts from ~ 0.68–0.47 and ~ 0.74–0.43 (Fig. 5). Because we do not find these families in the C genome, tetraploidization must have happened after they went silent. Thus, we propose that the tetraploidization which gave rise to *A. insularis* happened less than ~ 0.43 Myr ago. Conversely, we did not find copies of the *A. sativa* A genome specific subfamily *RLG_Ava_G3_4* in the C and D genome of *A. sativa*. Thus, we conclude that the hexaploidization (ACD) occurred no more than ~ 0.15 Myr ago, as the burst of this family ended around that time in the *A. sativa* A genome.

To complement the molecular dating analysis of polyploidization events, we included a C and D genome specific LTR retrotransposon family (*RLG_Aurora)* which is not centromere specific and belongs to the *Tekay* retrotransposon lineage [14]. *RLG_Aurora* is the only TE family that we found to have an activity burst specifically in the C and D subgenomes of *A. sativa* and *A. insularis* (i.e. it was active after the tetraploid formed and became silent before hexaploidization, Additional file 1: Fig. S14). Indeed, *RLG_Aurora* is found in all diploid genomes only at very low copy numbers but has ~ 1,000 copies in the tetraploid and the C and D subgenomes of the hexaploid. *RLG_Aurora* was most active between ~ 0.42 and ~ 0.35 Myr ago in the C and D genome. Using the IQR as a marker we estimated the burst to have happened from around 0.48 to ~ 0.24 Myr ago, which fits into the period between tetra- and hexaploidization estimated above, as this time of the burst is located within the estimated time window between the tetraploidization (CD) and hexaploidization (ACD). *RLG_Aurora* is thus one of the few documented examples of a TE family being activated after polyploidization.

Taken together, the comprehensive analysis of two centromere specific retrotransposon families strongly supports the hypothesis that *A. insularis* is the CD genome donor of hexaploid oats [31, 41]. For the *A. sativa* A subgenome, we conclude that its progenitor it is basal to *A. longiglumis* and *A. atlantica*, as the latter two share more similarities in their TE activity profiles and are therefore closer related to one another than to the *A. sativa* A subgenome. This is in line with the recently presented *Avena* subgenome phylogeny by Zhang and colleagues [51].

### *RLG_Ava* and *RLG_Cereba* lineages are conserved across the *Poaceae*

**Fig. 6:**
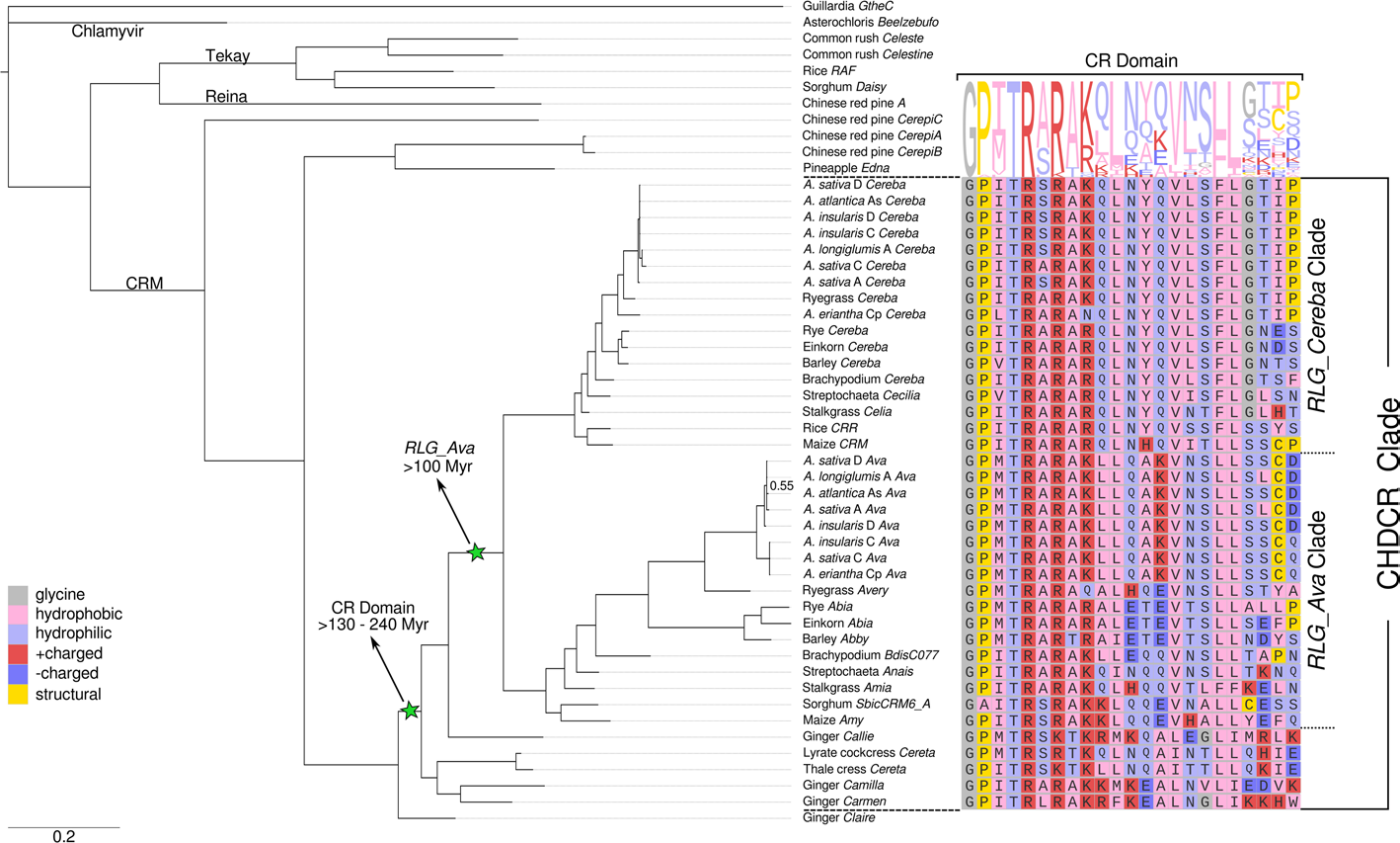
Phylogenetic tree of predicted proteins of *RLG_Cereba* and *RLG_Ava* and homologous TE families. The tree was built from the RH, RT and INT protein domains of the different TE families using MrBayes (generations = 235,000, burn-in of 25%). The green stars indicate the rise of the CR domain and the emergence of the *RLG_Ava* homologs in grasses (*Poaceae*). The *Guillardia theta* (GtheC) sequence was used and set as the outgroup in the MrBayes analysis. The CR-domain sequences with the conserved motive are displayed next to the respective consensus. Bipartition posterior probabilities below 0.8 are explicitly annotated

To further investigate the relationship between the two centromere specific family *RLG_Cereba* and *RLG_Ava*, we identified homologs in various grasses as well as more distantly related species such as ginger, pineapple, pine, the two *Arabidopsis* species *Arabidopsis thaliana* and *Arabidopsis lyrata*. In addition, we included sequences from other previously described retrotransposon lineages, such as *Tekay*, *Reina* and *Chlamyvir* [14]. We then constructed a phylogenetic tree from the predicted proteins of these TE families using MrBayes [63]. We found the homologs of *RLG_Ava* and *RLG_Cereba* to form two distinct clades within the phylogenetic tree. For each of the investigated grass genomes we found a homolog for both families, indicating that the two TE lineages must have been present in the ancestor of all grasses (*Poaceae*) before the divergence of *Streptochaeta* ~ 100 Myr ago [64] (Fig. 6). This indicates that the two families have been coexisting and apparently competing for the same genomic “niche”. The term competition is borrowed from ecology, where it describes interactions between organisms (in this context the two CRM families) who both require a specific limited resource. We regard the centromeres, i.e. the space where both CRM families integrate into, as such a limited resource, as centromere size is limited by factors such as CENH3 availability [24].

Interestingly, the *RLG_Ava* elements of the different *Avena* species cluster in an AD and a C genome clade while the *RLG_Cereba* elements from the different (sub-) genomes do not follow this pattern and are more similar to one another (Fig. 6).

When focusing on the CR-domain, we identified a clear pattern of conservation of the previously described RARA[AK]-motive (here R[AS]RA[RK]), especially of the positively charged amino acid positions which strengthens the hypothesis of an important role of this sequence motive in the centromere specificity of these elements recently presented by Heuberger et al. [20] (Fig. 6). Further, we found this motive to be conserved not only across the *Poaceae* but further in ginger and interestingly also in the dicot *Arabidopsis*. In the *RLG_Claire* element from ginger we were not able to detect a CR-domain (Fig. 6). However, despite detailed analysis we cannot exclude the possibility of an unresolved frameshift due to suboptimal quality of the consensus, causing this domain to not be detected. Most of the analyzed sequences displayed in Fig. 6 without a CR-Domain, contain a CHROMO (CHRomatin Organization Modifier) domain, which we identified using NCBI conserved domains. Merely the *RLG_CerepiC* (identified in Chinese red pine) and *RLG_Daisy* (identified in *Sorghum*) families did not exhibit this additional domain.

As we find the CR-domain present in phylogenetically distant plants, its origin must lie in the common ancestor of monocotyledons and dicotyledons more than 130 to 240 Myr ago [65, 66]. To investigate the origin of the CR-domain we built a hidden Markov model (HMM) to search against *Arabidopsis* proteins (TAIR v10.1). However, we were not able to identify a protein with a similar domain, indicating that the CR domain did not evolve from a known plant protein. Further, a search against the NCBI CD database did not yield a result.

Although there are instances in which the CR domain is missing from CRM retrotransposons, for example in CRM (maize) group B elements [18], the conservation of the CR-domain, specifically the positively charged amino acids of the R[AS]RA[RK]-motive, for a span of over 100 Myr greatly emphasizes its importance for the centromere specificity of TEs.

## Discussion

In this study, we analyzed centromere sequence composition and organization in multiple *Avena* species. Hexaploid oat has one of the largest genomes sequenced so far, and centromeres in large-genome crops are notoriously difficult to sequence [5, 25, 26]. The more relevant it was that we had access to high-quality genome assemblies from several species of the *Avena* genus. On the one hand, this allowed for in-depth analysis of individual centromeres, and on the other hand, for comparative analyses of centromeres between species.

In general, we found that functional centromeres largely coincide with the insertion sites of centromere-specific retrotransposons. This mirrors previous studies in wheat [5, 25], which showed that the youngest centromere-specific TE copies are mostly found inside or close to the functional centromeres. One limitation of this study is that we did not have CENH3 ChIP-seq data from the *A. sativa* accession and relied on other studies to predict positions of functional centromeres. However, previous studies showed insertion ages and distributions of centromere-specific TEs can be used as markers of current and past centromere positions [5, 55].

The *Avena* centromeres of the A and D subgenomes are mostly composed of three LTR retrotransposon families, *RLG_Cereba*, *RLG_Ava* and *RLG_Beth*. *RLG_Cereba* and *RLG_Ava* are putatively autonomous TEs which encode all proteins necessary for their replication as well as a CR domain that presumably guides insertions to functional centromeres through targeting of centromeric CENH3 histone variants [20]. In addition, we found that *RLG_Beth* is a non-autonomous element that is likely cross-mobilized by *RLG_Ava*. This is surprisingly similar to the autonomous/non-autonomous pair *RLG_Cereba* and *RLG_Quinta*, which shape the centromeres in Einkorn wheat [20]. However, *RLG_Beth* and *RLG_Quinta* have no sequence homology and thus must have evolved independently.

Despite these similarities, we made several findings that distinguish *Avena* centromeres from each other and from previously described plant centromeres, emphasizing the importance of comparative analyses for evolutionary studies. These findings are discussed below.

### Two retrotransposon families apparently compete for the centromeric niche in *Avena* and its relatives

Our phylogenetic analysis showed that the evolutionary *RLG_Ava* and *RLG_Cereba* lineages had been present already in the common ancestor of the grasses (*Poaceae*), which dates their origin to ~ 100 Myr ago [64]. Thus, the two retrotransposon lineages must have coexisted in grasses since then. Both of them are centromere specific, i.e. insert close to CENH3 marks, which makes the available space within the functional centromere a limiting resource. Indeed, previous studies suggest that the amount of CENH3 may determine the centromere size [24]. Thus, mirroring the concept of competition from ecology, we argue that their relationship can be described as competing for the centromeric “niche”. Interestingly, our data indicate that the dynamics can change rapidly in evolutionary terms: the *Avena* C genomes are practically void of *RLG_Cereba* elements, meaning that they had become largely silent after the C genome lineage diverged from the A/D lineage ~ 8 to 10.9 Myr ago [33, 51]. This is similar to findings in the Triticeae tribe (wheat, barley and rye) where the *RLG_Ava* lineage went largely silent, as mostly fragments of *RLG_Abia* (the *RLG_Ava* homologs) were found in wheat [20]. In contrast, the *Avena* A and D genome lineages show recent activity of both *RLG_Ava* and *RLG_Cereba*, indicating that both can persist simultaneously at similar abundance in a given species. This raises the question why none of them went completely extinct in a given species or species groups and how they were able to persist over long evolutionary times. Even if one of them goes largely silent, a few functional copies must be maintained, allowing re-emergence of the TE family at a later point in time. Another possibility is that there is occasional horizontal transfer (HT) of CRM retrotransposons between species. Indeed, multiple studies suggested that HT might be frequent, especially between closely related species [67]. This may allow TEs to circumvent silencing mechanisms (e.g. through small interfering RNAs) that were established in a given species [68]. However, we did not find any of the phylogenetic incongruencies that are defining characteristics of HT and therefore see it more likely that the respective TE families persisted at low abundance.

### Retrotransposon populations shed light on the evolutionary history of *Avena* species

Molecular dating of TE activity bursts showed that different retrotransposon subfamilies emerged and were active at different times and in different *Avena* genomes. This informed us on the relationship between *Avena* genomes and the timing of divergences and polyploidization events. For example, the *Avena* A genomes contain multiple lineage-specific subfamilies of *RLG_Cereba* and *RLG_Ava* retrotransposons that were highly active in very recent evolutionary times, giving individual A genome species highly distinct TE profiles. This showed that the A genome of *A. sativa* is clearly distinct from both the other investigated A genomes species *A. longiglumis* and *A. atlantica*. In general, our results support those from previous studies on the phylogeny of the *Avena* genus [31, 33, 51] with respect to species divergence times. Here, we want to emphasize that for all our molecular dating procedures, we used the nucleotide substitution rate of 1.3E-8 per site per year, which is typically used to date insertion ages of retrotransposons [69]. Our estimates for species divergences tend to be slightly more recent than those done with other methods. For example, we place the divergence of *A. atlantica* and *A. longiglumis* from the A genome of *A. sativa* at ~ 1.2 Myr, while recent studies placed this divergence at ~ 1.7 Myr [46] and ~ 2.8 Myr [33], respectively.

The main differences we found in the dating of polyploidization events. Previous studies dated tetraploidization to between 1.5 and 4.0 Myr ago and hexaploidization to 0.4—0.8 Myr ago [51] and ~ 2.8 Myr [33]. In contrast, our data on presence/absence of TE (sub-) families and TE activity bursts indicate that tetraploidization and hexaploidization could have occurred as recently as 0.43 and 0.15 Myr ago, respectively. Thus, analysis of TE populations and TE bursts could provide complementary information for future studies to narrow down the timing of these polyploidization events.

Interestingly, analysis of *RLG_Aurora* (a retrotransposon family that is not centromere-specific) indicated that there must have been a time span of ~ 300,000 years between formation of the tetraploid and the hexaploid, because *RLG_Aurora* had an activity burst that is found exclusively in the tetraploid (CD) and the C/D subgenomes of hexaploid oat. This family must have been largely silent before tetraploidization, as well as at the time when the hexaploid formed. Thus, *RLG_Aurora* might be a rare remnant of a “genomic shock”, a dramatic activation of TEs, that is postulated to follow polyploidization [70, 71]. However, no evidence for genomic shock was found in other polyploids such as wheat [19], nor did we find any similar TE activity burst following hexaploidization.

### *Avena* centromeres show evolution of different centromeric architectures

One of the most curious findings of our study were the strikingly different centromere architectures in the A, C and D genome lineages. It has long been known that plants have a variety of centromere architectures [72]. In *Arabidopsis*, for example, centromeres largely consist of short tandem repeats that are occasionally interspersed with TEs [73], while in maize and wheat, they are largely comprised of CRM retrotransposons [5, 13]. Intermediate forms that contain both TEs and tandem repeats in abundance are, for example, found in *Brachypodium* [6]. Nevertheless, we were surprised to find such variety in a genus that diverged probably only 8 to 10.9 Myr ago [33, 51].

While the centromeres of the A and D genomes are nearly exclusively comprised of LTR retrotransposons of the *RLG_Cereba*, *RLG_Ava* and *RLG_Beth* families, those in the C genomes lack *RLG_Cereba*, but contain a high number of tandem repeats. Furthermore, the three lineages show very different TE activity profiles: the A genomes contain multiple TE subfamilies that were very recently active, and may, in fact, still be active now. In contrast, the D genomes contain multiple subfamilies that were highly active within the past million years but seem to be silent currently. Furthermore, only one of the identified subfamilies (*RLG_Cereba_G1_6*) was found in both the A and D genomes. This indicates that centromere compositions can vary strongly even in very closely related species.

However, the most dramatic differences were found in the *Avena* C genomes. Not only do *RLG_Cereba* elements seem to have gone nearly extinct, the *RLG_Ava* retrotransposons have been largely silent for at least the past million years. Additionally, we found that ~ 19.6% of centromeric sequences are composed of tandem repeats. While a previous study reported the centromeric tandem repeats Cen48 and Cen87 [30], we find these sequence motifs in hundreds of thousands of copies across the entire C genome and therefore propose to rename them ATR48 and ATR87. These highly repetitive sequences must have emerged in the C genome ancestor, as they are found in high abundance in *A. sativa*, *A. insularis* and *A. eriantha*. Similarly, He et al. described two centromeric repeats (Cen46 and Cen55) in *A. sterilis* [32].

We have no hint as to the origin of these sequences since they show no homology to known TE sequences. Neither do we know whether the emergence and spread of these tandem repeats have any causal connection to the silencing of the CRM *RLG_Ava* retrotransposons. However, our data indicate that CENH3 deposition in the C genome is less strictly associated with insertion sites of the youngest CRM retrotransposons than in the A and D genomes. This could suggest that other (e.g. epigenetic) factors influence positioning of centromeres in the C genome. However, we acknowledge that we projected the position of the centromeres in *A. sativa* OT3098 from CENH3 data of another accession [52]. This strategy can merely provide estimations, as recently demonstrated for the *T. aestivum* accessions Chinese Spring and Julius [25].

### On the origin of centromere-specific retrotransposons in grasses

All centromere-specific retrotransposons in grasses described so far are of the CRM-type which have in common a distinct CR domain fused to their integrase. Recent studies indicated that the CR domain may indeed interact directly with centromeric CENH3 histone variants and thus guide new insertion to the functional centromere [20]. It has long been known that TEs sometimes acquire protein domains from cellular genes, which gives them new functions. For example, a number of retrotransposons were shown to have acquired chromodomains which are known to recognize histones [22]. However, the origin of the CR domain is still unclear as it lacks homology to canonical chromodomains [14]. In this study, we searched plant protein databases with HMMs of the CR domain but did not find any homologs. One explanation is that the CR domain is so highly diverged that its origins are not detectable anymore. Alternatively, the CR domain could have been acquired through horizontal transfer from a different organism group (e.g. bacteria). However, our search of conserved domains at NCBI also did not yield any results. It is still possible that the CR domain was acquired from an unknown source organism that is not represented in the conserved domain libraries, as previously suggested [14]. As a third possibility, we hypothesize that the CR-domain may represent a true evolutionary innovation. It could have been gained by chance through an extension of the canonical reading frame (i.e. through loss of a stop codon) in the *RLG_Ava* and *RLG_Cereba* ancestral TE, aligning with the general idea of a spontaneous acquisition recently proposed by Neumann and colleagues [14]. At this point we favor this last hypothesis.

## Conclusions

The high-quality assemblies of multiple genomes from the *Avena* genus allowed for detailed comparative analyses of centromeres and their evolutionary dynamics in these highly repetitive genomes. We showed that *Avena* centromeres are largely composed of two retrotransposon families but also found striking differences in centromere architecture and sequence composition. These included the emergence and disappearance of retrotransposon families and subfamilies, inversions and the spread of novel satellite repeats. Considering that the different species studied here all belong to the same genus and are closely related, our data show that centromeres are highly dynamic and can rapidly take different evolutionary trajectories. Thus, continuing to produce high quality assemblies especially from large and complex crop genomes will provide valuable resources to investigate the molecular mechanisms that drive centromere evolution on a broader evolutionary scale.

## Methods

### TE annotation

The initial TE annotation was performed by running EDTA (v2.2.1 [59]) on the OT3098 v2 centromeres using the settings --overwrite 1 --sensitive 1 --anno 1 --force 1. A subset of the TREP database v.19 [74] was used as a curated library input and further was utilized to manually annotate the identified high copy sequences on a family-level by using them as a query in a blast search against the database. We provide a curated library combining specifically Poaceae TEs filtered from the TREP database [74] and grass TEs which were manually curated during the course of this study on GitHub. After preparing consensus sequences for the TEs annotated by EDTA with high abundance (see below “Construction of consensus sequences”), we ran EDTA again, this round with an updated curated library, which included all produced consensus sequences and thus provided a more comprehensive annotation of the centromeric region of *A. sativa* (see Fig. 2).

### Construction of consensus sequences

The EDTA output (see above “TE annotation”) was used to manually curate consensus sequences by using the most abundant sequences provided by EDTA as a query input for a genome wide blastn search against the respective species. Hits with an identity of at least ~ 80% and length of at least 80% of the query sequence were extracted with flanking sequences from the respective genome. From this set we used a minimum of 40 randomly chosen sequences. These were aligned using clustalw with default settings and the alignment cut on the start and end site of the TEs. From this cut alignment, the initial consensus sequence is derived. This initial consensus was used in the TEpop pipeline (see below). Using a blastn search against the TREP database, we identified known families. For each of the four families *RLG_Cereba, RLG_Ava*, *RLG_Beth* and *RLG_Aurora*, we also constructed consensus sequences for the entire families by using 40 randomly picked copies from all full-length TEs isolated with the TEpop pipeline. The 40 copies were aligned with clustalw using a gap creation penalty of 10 and a gap extension penalty of 0.2, from which the consensus was then derived. Consensus sequences represent individual copies extremely well, as in all cases > 99% of the length of the consensus can be aligned with individual copies (Additional file 1: Fig. S15).

In addition, we derived consensus sequences in the same way for all subfamilies defined in the TEpop/PCA analysis described above. The same was done for *RLG_Abia* and *RLG_Cereba* families from Triticeae using previously published datasets from wheat [25], *Triticum monococcum* [5], barley [26] and rye [18].

In all cases, DNA consensus sequences contained a single open reading frame (ORF) encoding the canonical RT/RH and INT domains. These hypothetical proteins were then used for the phylogenetic analysis described below. In addition, we derived consensus sequences in the same way for all subfamilies defined in the TEpop/PCA analysis and the consensus sequences spanning the *Avena* genus which were used in the TEpop analysis described below. All consensus sequences as well as fasta files for all isolated full-length copies were deposited on our Github page [75]. Only manually curated consensus sequences were used in this study. The lineage of the TE families was identified by using the consensus sequences as a query for blastx searches against the REXdb database [14].

### TE population analysis

Following the pipeline described in Wicker et al. [53], full-length elements were identified starting with the isolation of around 100 LTRs per family and genome using the initial consensus sequences (see above). Next the LTRs were aligned using Clustalw with a gap opening penalty of 10 a gap extension penalty of 0.2. If variants of LTRs were visible as groups in the alignment, group specific consensus sequences were constructed. These consensus LTRs were then used as queries for blast sequences against the chromosomes of *A. sativa* (69 for *RLG_Ava*, 11 for *RLG_Cereba*, 45 for *RLG_Beth* and 5 for *RLG_Aurora*). For all of these LTR consensus sequences the following searches were performed: If two LTRs were found in the same orientation, within a range of ± 2000 bp of the original consensus and not more than 5 bp were missing from either of the LTRs (checking for completeness), an identified element was classified as full-length. This was done with the script TEpop_RLX_2024. An additional filtering step for size was applied to remove elements with large insertions from deletions. In addition, blast searches against the TREP database [74] were performed to confirm the family identity (blast hits > 2500 bp, 80% identity). Further, we removed copies with gaps of more than 700 bp (*RLG_Cereba, RLG_Aurora*) and 950 bp (*RLG_Beth* and *RLG_Ava*, to account for differences in the LTR repeat clusters). This led to a clean data set in which the consensus sequence aligned over > 99% of its length with individual copies of the respective retrotransposon family (across the *Avena* genus) (Additional file 1: Fig. S15). The full-length TEs were then used in the following analysis. To estimate the insertion age of the individual TEs we used the scripts LTR and date_pair. They extract the LTRs and align them using the program Water from the EMBOSS package, distinguishes between transitions and transversions and applies a rate of 1.3E-8 per site and year for the molecular dating [54, 69]. For each insertion age estimate, the standard deviation is calculated. Since standard deviation for very young copies can be in the range of the age estimate itself (see Additional file 1: Fig. S16), we considered estimates > 100,000 years as reliable. Identical LTRs (resulting in an insertion age of zero) were also considered reliable.

We then applied the last layer of filtering by removing copies that had an estimated insertion age higher than the 95% quantile of the respective subgenome and family. The individual full-length copies were aligned against the consensus spanning the *Avena* genus (see construction process above) using the program Water (EMBOSS package) with a gap opening penalty of 50 and a gap extension penalty of 0.1. We used the script pair_to_vcf_2024 to combine the pairwise comparisons into one variant call format (vcf) file. Variants with an occurrence of less than 5% were filtered out (minor allele frequency set to 5%). To calculate the PCA we used the R packages SNPRelate (v1.38.0, [76]) and gdsfmt (v1.40.0, [76]).

To display the PCA for identification of the TE subfamilies visual_PCA_TEpop_2024 was used. The subfamilies were defined by visual inspection of the plot within the user interface following the TEpop pipeline. The 95th percentile of the estimated insertion age per unit (e.g., subgenome or identified subgroup) was used as a cut off for copies to include in further analysis of these groups as estimated insertion ages above this threshold are likely artefacts. For example, they can be the result of a crossing over of two TEs thus leading to pairing of two unrelated LTR sequences. In the following, these insertion ages were used for the divergence time estimation. For violin plots only groups with more than 10 copies were included. For the visualization we used R (R Core Team 2024) and the packages stringr (v1.5.1, [77]), ggplot2 (v3.5.1, [78]), dplyr (v1.1.4, [79]) and tidyverse (v2.0.0, [80]). For *A. sativa* C0648 and *A. sterilis* we utilized version v2 of the program date_pair, version v1 (different output format) was used for all other genomes. All scripts used can be found on GitHub [81] and through Zenodo [82].

### Comparison of substitution rates of retrotransposons families

Using insertion age estimates of multiple retrotransposon families raised the question whether substitution rates are similar in different families, thus allowing direct comparisons. Higher mutation rates could, for example, result from a higher error rate of the reverse transcriptase, or from a higher methylation rate in one TE family (resulting in more C- > T and G- > A substitutions). To study substitution rates, we compared the youngest insertions (insertion age < 50,000 years) in *A. sativa* and *A. longiglumis* of *RLG_Cereba* (24 and 38 copies, respectively) and *RLG_Ava* elements (18 and 73 copies, respectively). All copies from a respective species and TE family were compared pairwise all against all using the program WATER (EMBOSS package, ubuntu.com). We argue that the pairs with the highest levels of sequence identity are those that are derived from the same active “master copy”. If a TE family or species has higher mutation rates, these pairs should show lower levels of sequence identity. We found distinct groups of highly identical pairs (presumably derived from the same active “master copy”) but found no differences in sequence identity levels between TE families or between species (Additional file 1: Fig. S17).

Additionally, we analyzed the frequencies of C to T and G to A substitutions (indicative of methylation levels) in the *RLG_Cereba* and *RLG_Ava* families. Here, we produced a vcf file for the youngest copies, analogous to the one that was done for the entire TE populations (see above). We then determined the fraction of C to T substitutions for both families and found nearly identical values (Additional file 1: Fig. S17b). From these results, we concluded that it is legitimate to directly compare insertion age estimates of the studied TE families.

### Comparison of removal rates of retrotransposons families

To analyze whether there is a bias in removal rate of individual TE families between species, we compared centromeric and peri-centromeric regions from the D subgenome of *A. sativa* and *A. insularis*. The procedure and results are shown in Additional file 1: Fig. S18. In a first step, chromosomes were aligned globally with the custom Perl script blast_compare_chromosome to identify colinear regions around centromeres. The selected collinear regions ranging from 5–30 Mb from chromosomes 1D, 2D, 4D, 5D and 6D were then aligned to higher resolution with blast_compare_chromosome. Insertions/Deletions (InDels) between 3 and 17 kb in size were identified in a semi-manual way with the custom script TE_PAP_from_log_chr_comp. The identified 666 InDels were then manually curated and classified into TE insertions and deletions with the custom script png_blast_coverage_TE_all. Insertions were defined as a sequence that is present in only one species that corresponds to a full-length TE copy including target sited duplications. All scripts and input data are deposited in our GitHub repository [75] and Zenodo [83, 84], along with a detailed description on how to use the Perl scripts and how they work.

### Centromere position estimation

Centromere positions were inferred using ChIP-seq dataset for hexaploid oat (*A. sativa* cv. Starter) [52]. We mapped the reads onto the OT3098 assembly using bowtie2 (v2.5.4, [85]) and filtered for multimappers using the view function in samtools (v1.6, [86]). Next, we ran bamCompare (deeptools v3.5.1, [87]) with a minimum mapping quality of 30 and a binsize of 500,000. From this bedgraph file we calculated a chromosome specific threshold of 25% of the maximum coverage. Next, we identified the bins which exceeded this threshold, as well as the last bin before the threshold was first met and the bin after the threshold was last met. Next, we inferred the start and end positions by using the Intercept Theorem to calculate the position on which the coverage of reads reached this threshold, while assuming linear increase between the bins. For these calculations, we utilized the starting positions of the bins. The bedgraph file and code for the calculation can be found on GitHub [75].

### Phylogenetic analysis of centromere specific retrotransposon consensus sequences

For species outside of the *Avena* genus and the Triticeae tribe, we relied on consensus sequences previously deposited in the TREP database [74]. From consensus sequences, hypothetical proteins were derived for phylogenetic analyses (see above “Methods” section “Construction of consensus sequences”).

To ensure that consensus sequences reliably represent the individual families and species, we randomly picked 10 TE copies which encode a presumably intact polyprotein (i.e. it encoded the RT/INT domains without frameshifts or in-frame stop codons) from our *Avena* sequences, from wheat, *T. monococcum*, barley and rye for both *RLG_Ava* and *RLG_Cereba* homologs. These predicted proteins, together with the respective consensus sequences were used for construction of a phylogenetic tree using MrBayes as described below. In all cases, the individual copies clustered together with their respective consensus sequences (Additional file 1: Fig. S19). From this, we concluded that the use of protein consensus sequences was appropriate for phylogenetic analysis of the TE lineages studied here.

First, we used ClustalW [88] to align the consensus sequences and created a cropped alignment using an in-house script. For the following analysis we used a subset of the full-length polyproteins including the RT, RH and INT protein domains, whose position was estimated beforehand using NCBI conserved domain batch search with standard settings. We used ClustalX [88] to transfer the alignment into nexus format and ran MrBayes [63] 25,000 generations with a burn-in value of 25% until the average standard deviation of split frequencies decreased to under 0.01. The data visualization was done using FigTree (v1.4.4, [89]) and R (R Core Team 2024) with the packages stringr (v1.5.1, [77]), ggplot2 (v3.5.1, [78]), ggmsa (v1.3.4, [90, 91]), ape (v5.8, [92]), ggtree (v3.10.1, [91]), ggtreeExtra (v1.14.0, [93]).

### Phylogenetic analysis of individual TE copies

We randomly selected 250 copies per subgenome (or as many as there were available) for the phylogenetic trees. The copies were aligned using MAFFT (v7.526) with the following parameters: --maxiterate 1000 --reorder --nomemsave --leavegappyregion -−6merpair --auto --reorder. Next, using RAxML-NG (v1.2.2) we ran the command raxml-ng --msa [alignment] --model GTR+G --seed 2 --threads 8 --tree pars{10},rand{10} --prefix [x] which ‘alignment’ indicating the alignment file created by MAFFT and ‘x’ indicating the prefix for output files. Next, we performed a bootstrap analysis on the best tree: raxml-ng --msa [alignment] --model GTR+G --seed 2 --threads 8 --bootstrap --prefix [y] --tree [x].bestTree --bs-trees 500. Lastly, by running raxml-ng --support --tree [x].bestTree --bs-trees [y].bootstraps --prefix [map_bootstraps] --threads 4 we added the bipartition to the best tree. Data on insertion age were derived from the TEpop pipeline ([53], see above). The final tree files with the bootstrap value information are deposited on GitHub [75] and Zenodo [83, 84].

### Hidden Markov models (HMM) for identification of CR-domain origin

To search for proteins similar to the CR-domain we build an HMM from an alignment of the sequences which were also used to build the phylogenetic tree, cropped to the CR-domain. Using HMMER (v3.4 (2023), [94]) we built the model with the function hmmbuild and searched the proteins of *A. thaliana* Tair v10.1 using hmmsearch.

### Tandem-repeat analysis

The putative centromeres were then annotated with tandem repeats finder (trf, [60]) using the settings: Match = 2, Mismatch = 7, Delta = 7 PM = 80, PI = 10, Minscore = 50, MaxPeriod = 2000. For whole genome annotation of the two tandem repeats ATR48 and ATR87 the query sequences were used for blastn searches and blast hits were filtered for 80% identity to the query and 40 bp or 80 bp alignment lengths respectively.

## Supplementary Information

Additional file 1. Supplementary information for “Transposable element dynamics drive rapid evolution of centromere architecture in the Avena genus”. This file contains supplementary figures S1 to S19 and supplementary tables Tab. S1 and Tab. S2

## Data Availability

The datasets analyzed during this current study are available in the GrainGenes repository (*A. sativa* cv. Sang v1.1 [31, 95];* A. sativa* OT3098 v2, PepsiCo [29], the NCBI Genome Database (*A. longiglumis* CN58138 v1 [31, 96], *A. eriantha* BYU132 v1 [50, 97], *A. insularis* BYU209 v1 [31, 98], *A. atlantica* CC7277 v1 [50, 99], *Arabidopsis thaliana* TAIR v10.1 [100], *A. sativa* C0648 [101], *Hordeum vulgare* [26, 102], *Secale cereale* [18, 103], *Triticum monococcum* [5, 104], *T. aestivum* [105, 106], *Pinus tabuliformis* [107], *Arabidopsis lyrata* subsp. *lyrata* [108, 109], *Zingiber officinale* [110], *Ananas comosus* [111, 112], *Brachypodium distachyon* [113], *Streptochaeta angustifolia* [114, 115], *Oryza sativa* [116], *Zea mays* [117], *Pharus latifolius* [118, 119]), the Genome Warehouse (*A. sterilis* [120]) and the Genome Sequence Archive (ChIP-seq dataset *A. sativa *cv. Starter [121, 122]). The produced TE consensus sequences were deposited in the TREP database [74, 83]. Utilized scripts for the TEpop analysis can be accessed through GitHub and Zenodo [81, 82]. The collection of TEs and code can be found on GitHub [75] and Zenodo [83, 84]. All data available through Zenodo is licensed under Creative Commons Attribution 4.0 International. All data and scripts available through GitHub are licensed under GNU General Public License v3.0.
