# Supplementary material for: Transposable element dynamics drive rapid evolution of centromere architecture in the Avena genus
Source: Genome Biol. 2026 Jun 16;27:200. doi: 10.1186/s13059-026-04127-6 (PMC13270711; doi:10.1186/s13059-026-04127-6)
Supplement: Supplementary file 1 — Additional file 1. Supplementary information for “Transposable element dynamics drive rapid evolution of centromere architecture in the Avena genus”. This file contains supplementary figures S1 to S19 and supplementary tables Tab. S1 and Tab. S2 [file 13059_2026_4127_MOESM1_ESM.pdf]

Supplementary information for "Transposable element dynamics drive rapid evolution of centromere architecture in the *Avena* genus"

Supplementary Figures

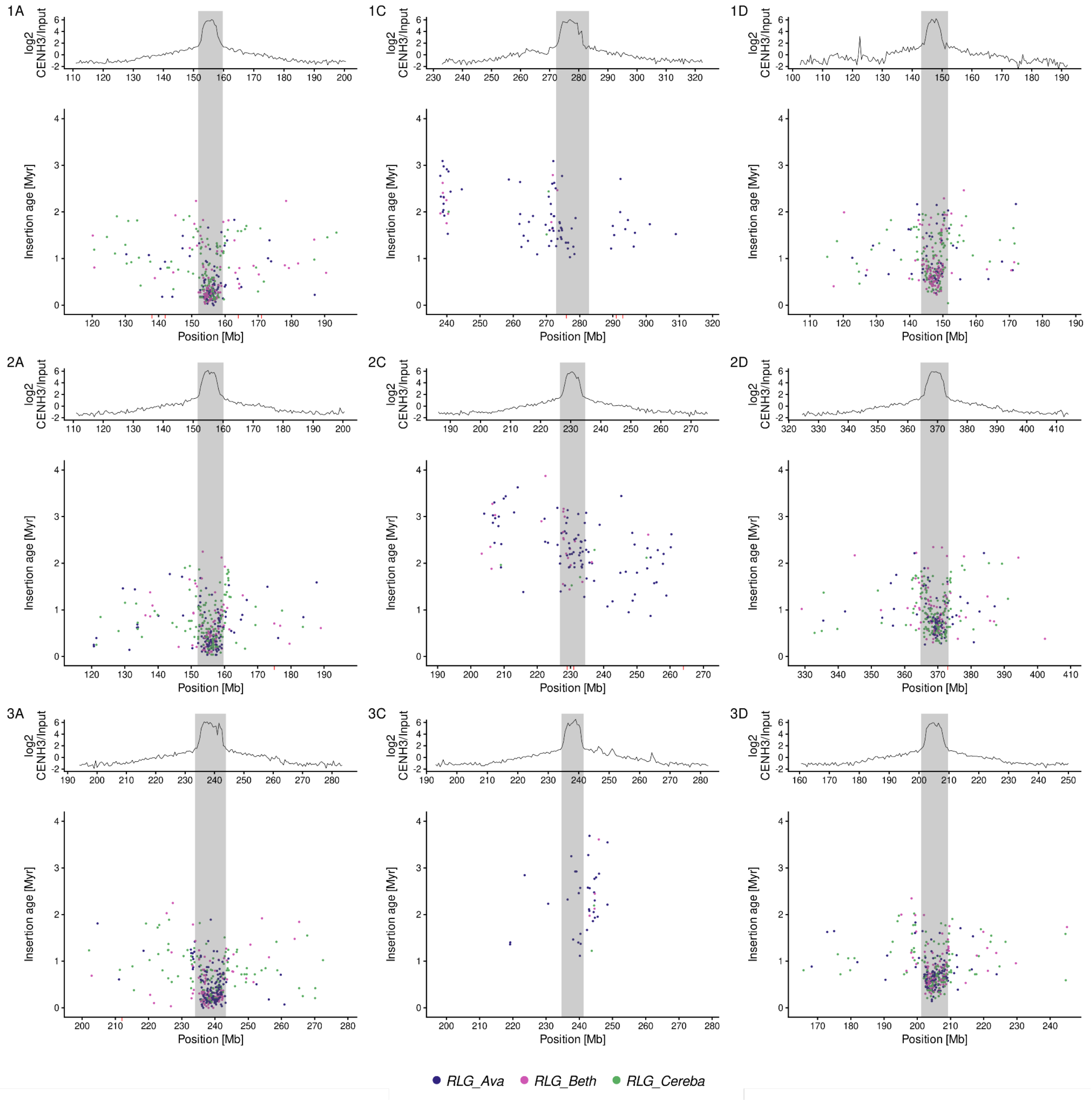

Fig. S1. CENH3 ChIP-seq data (top, binsize 500kb) and identified full-length copies of the centromere specific families *RLG_Ava* (violet), *RLG_Beth* (pink) and *RLG_Cereba* (green) in the (peri-) centromeric region of *A. sativa* OT3098 for each chromosome (bottom). The gray boxes indicate the centromere positions inferred from CENH3 ChiP-seq data. Copies with an estimated insertion age of under 4 Myr are shown. Note the estimated insertion ages of these TE families are older in the C subgenome and the copies more dispersed in comparison to the other two subgenomes. In the A and D subgenome the

TEs overall show lower insertion age indicating more recent activity. Red marks under the x-axis indicate the assembly gaps.

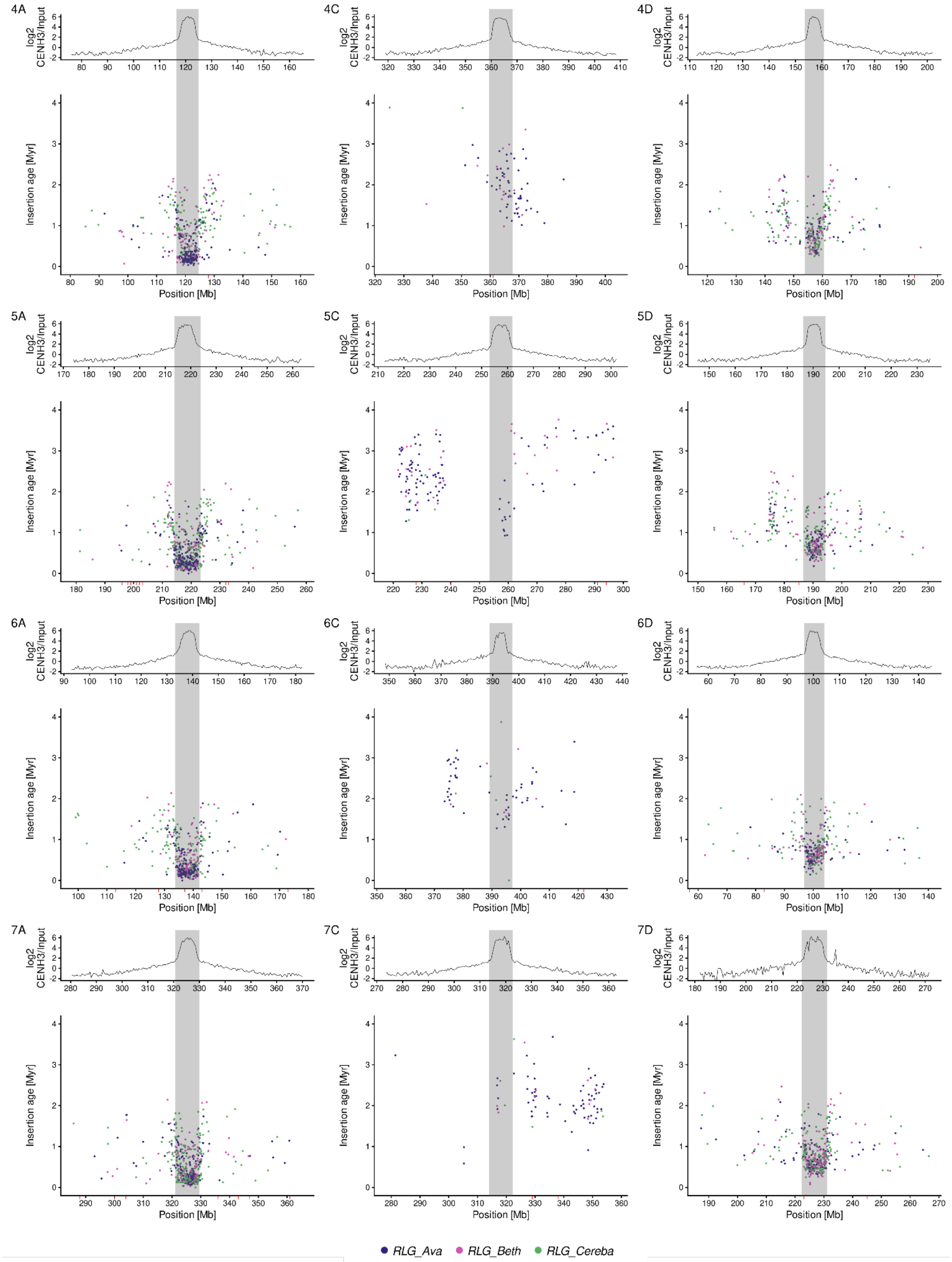

Fig. S1. continued.

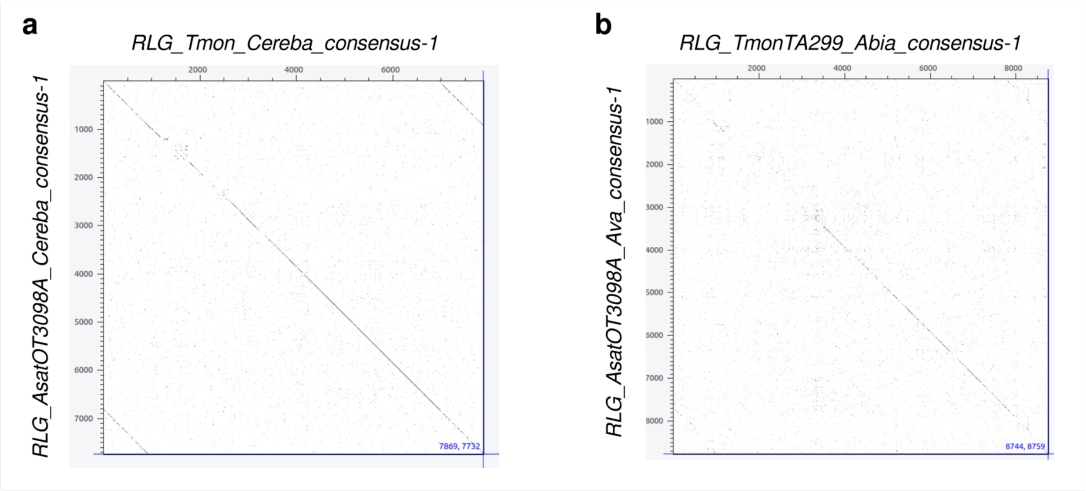

Fig. S2. Examples of dot plot comparisons between *RLG_Cereba* **(a)** and *RLG_Abia / RLG_Ava* **(b)** from *T. monococcum* and *A. sativa* OT3098.

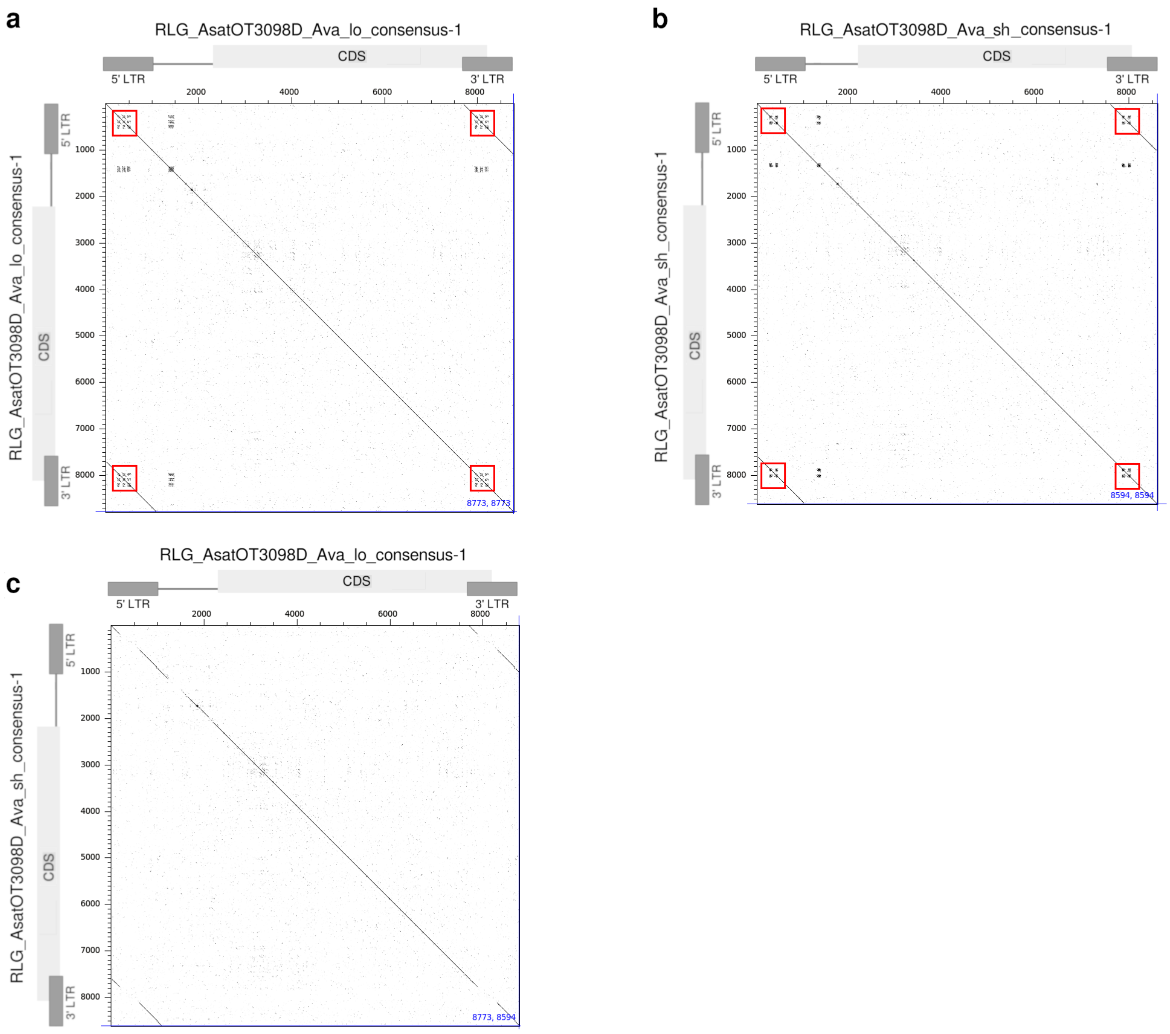

Fig. S3. Dot plot comparisons of variants of *RLG_Ava* retrotransposons. **(a)** Dot plot alignment of the longer variant RLG_AsatOT3098D_Ava_lo_consensus-1 against itself. *RLG_Ava* elements contain small tandem repeat clusters in their LTRs (indicated by red boxes) which differ among the main variants. **(b)** Dot plot alignment of the shorter variant RLG_AsatOT3098D_Ava_sh_consensus-1 against itself. **(c)** Dot plot alignment of the two variants. Note that the tandem repeat arrays show no DNA sequence conservation while the rest of the sequence is well conserved.

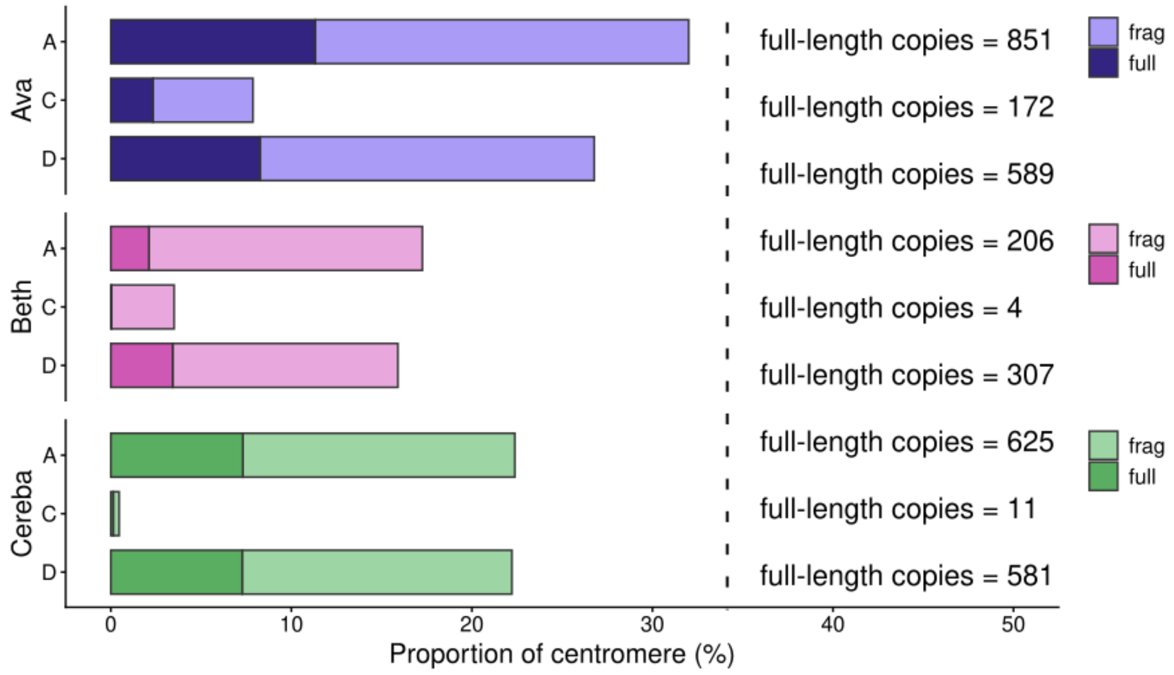

Fig. S4. Fraction of full-length copies of total annotation TE sequence per family in centromeres based on EDTA annotations. (frag: fragmented)

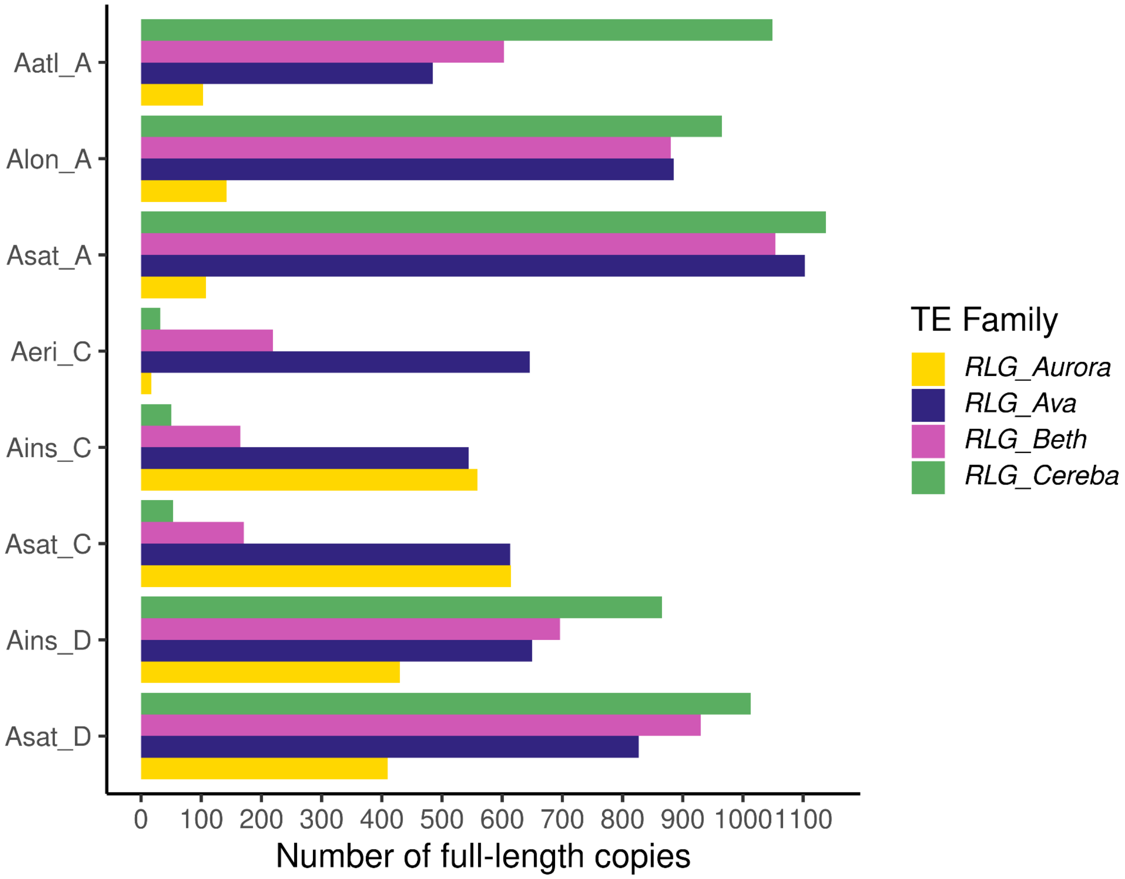

Fig. S5. Number of identified full-length copies of *RLG_Ava*, *RLG_Beth*, *RLG_Cereba* and *RLG_Aurora* in different genomes.

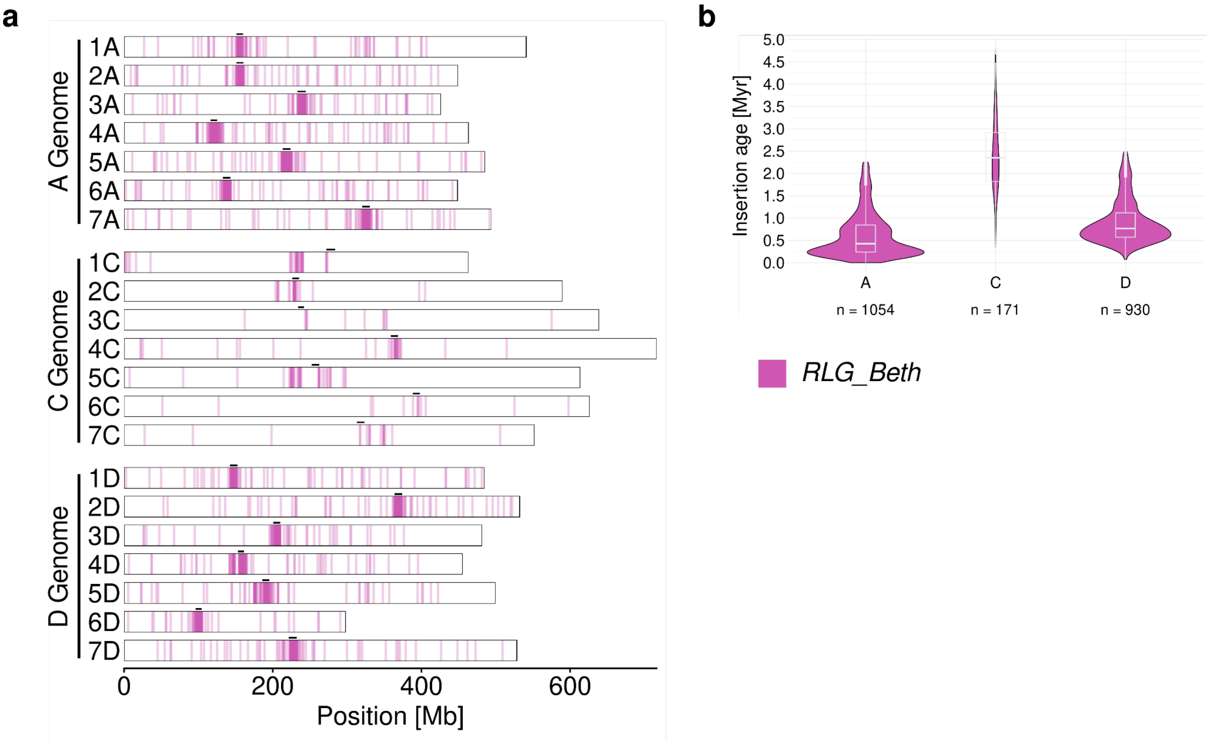

Fig. S6. a. Localization of full-length copies of *RLG_Beth* retrotransposons across the *A. sativa* OT3098 genome. Centromere positions are indicated by horizontal black bars. **b.** Violin plots of *RLG_Beth* copies' estimated insertion age.

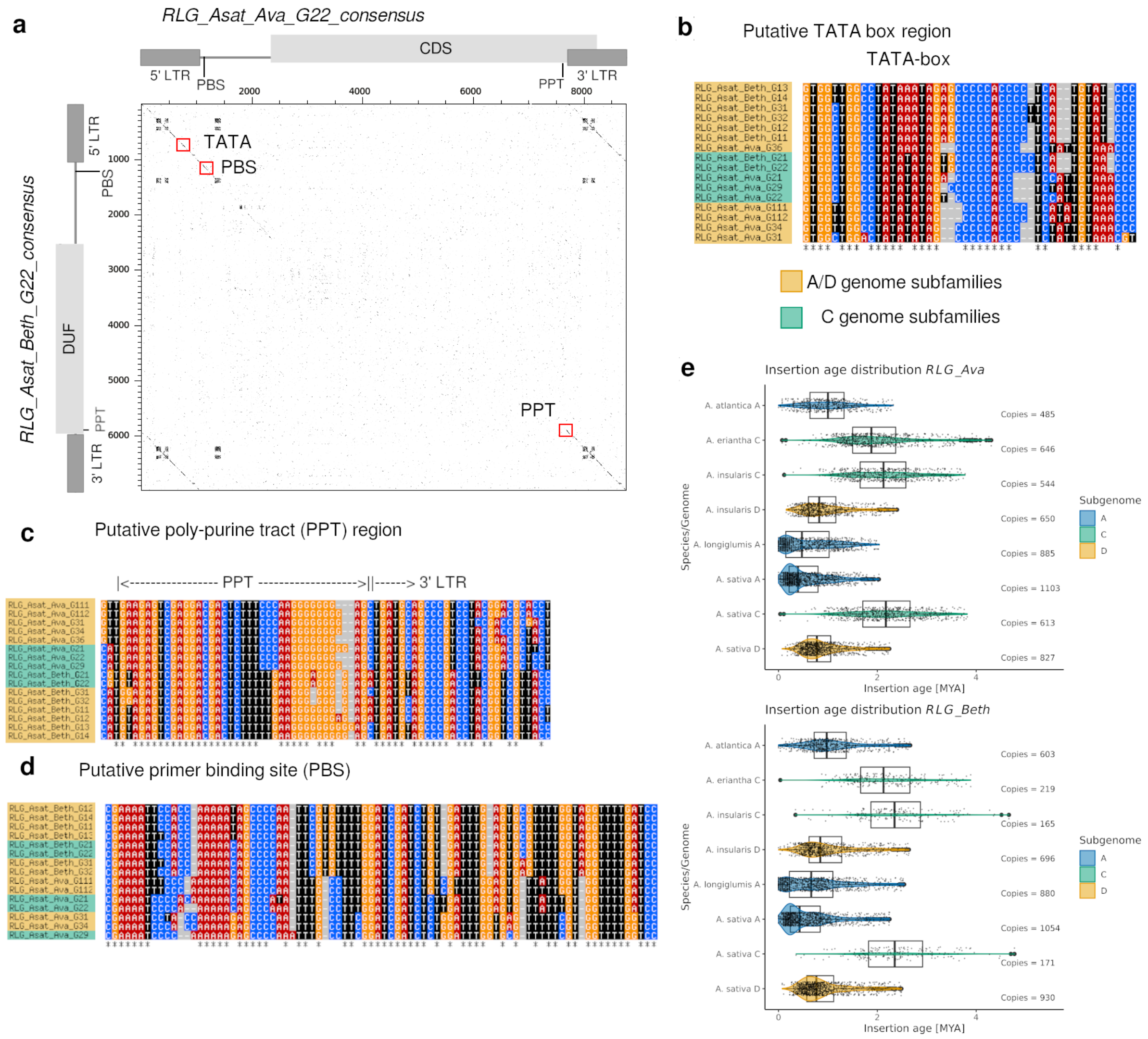

Fig. S7. Comparison of *RLG_Ava* and *RLG_Beth* retrotransposons indicates that *RLG_Beth* is a non-autonomous family that is mobilized by *RLG_Ava*. **a.** Dotplot comparison between consensus sequences for subfamilies *RLG_Ava_G2_2* and *RLG_Beth_G2_2*. *RLG_Ava* encodes the typical retrotransposon proteins necessary for replication, such as reverse transcriptase, integrase etc. while *RLG_Beth* only encodes a domain of unknown function (DUF). Parts of LTRs such as a putative TATA box regions, as well as diagnostic motifs such as primer binding site (PBS) and poly-purine tract (PPT) are conserved between the two, suggesting that the autonomous *RLG_Ava* retrotransposons cross-mobilize the non-autonomous *RLG_Beth* elements. **b.** Multiple alignment of the putative TATA-box containing region from representative subfamilies. **c.** Multiple alignment of the putative PPT region. **d.** Multiple alignment of the putative PBS region+. Note that *RLG_Ava* and *RLG_Beth* subfamilies from the same subgenome(s) share sequence motifs. Additionally, shared motifs between *RLG_Ava* and *RLG_Beth* suggest recombination between the two. **e.** Insertion age distribution of *RLG_Ava* and *RLG_Beth*. Note that *RLG_Ava* and *RLG_Beth* show very similar insertion age distributions, indicating that they were active at the same time and at similar levels.

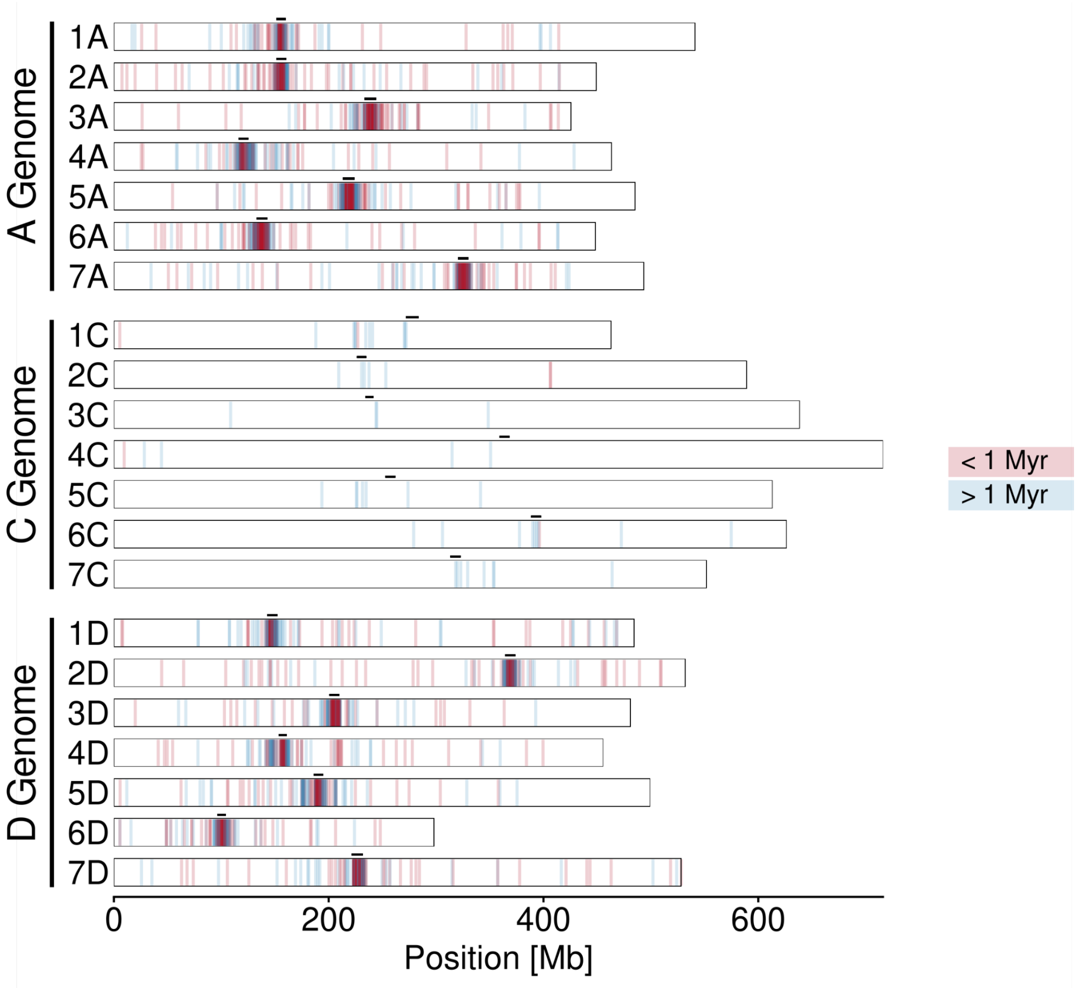

Fig. S8. Full-length *RLG_Cereba* elements in *A. sativa* OT3098 colored by age groups, young elements within insertion ages under 1 Myr are displayed in red and older elements with insertion ages over 1 Myr are displayed in blue. Black bars indicate the projected centromere positions.

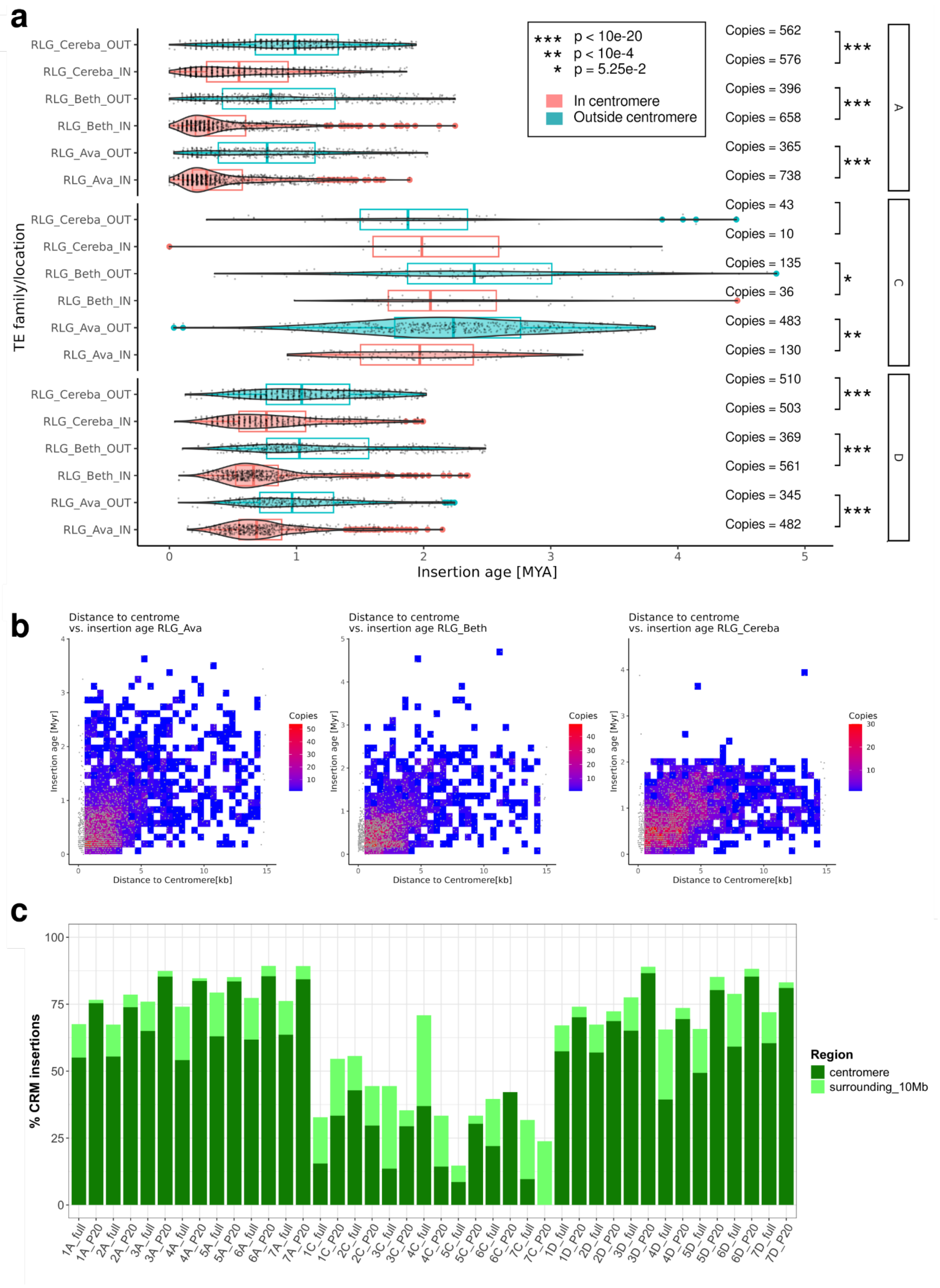

Fig. S9. Comparison of insertion ages of centromere-specific TEs inside and outside of the predicted centromeres. **a.** All identified full-length TE copies were grouped based on whether they lie inside or outside of predicted centromeres. Additionally, TEs from the three subgenomes were analysed

separately. Differences between TE copies from inside and outside the centromere were tested with the R wilcox.test package (one-sided test with the parameter alternative=”less” for insertion ages of TE inside centromeres). **b.** Plot for correlation between insertion age and distance to the midpoint of the predicted centromeres for centromere-specific TEs. The datapoints for the individual TE copies are plotted a gray dots and density is plotted as head map. Note the strong correlation between insertion age and distance from the centromere. **c.** Comparison between all extracted insertions of *RLG_Cereba*, *RLG_Ava* and *RLG_Beth* (full) and those with insertion ages at or below the 20th percentile per chromosome (P20) and their localisation. The dark green indicates insertions within the centromere boundaries, while the light green highlights the CRMs located in the 10Mb to both sides of the centromere (+/-10Mb). Note the P20 subset consistently shows higher a percentage of CRMs located within centromere boundaries in the A and D subgenome in comparison to the full dataset, i.e. the youngest elements are reliable markers for the centromeric region in these two subgenomes.

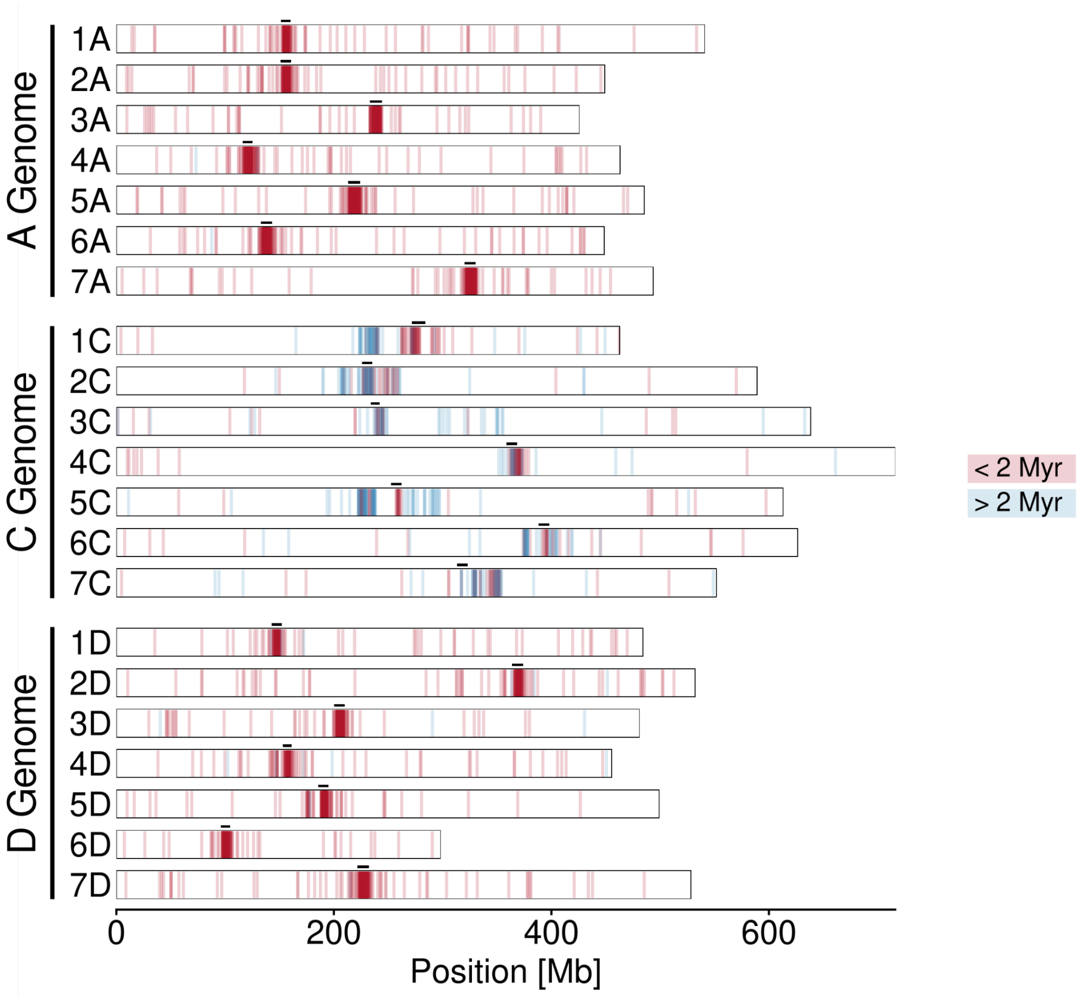

Fig. S10. Full-length *RLG_Ava* elements in *A. sativa* OT3098 colored by age groups, young elements within insertion ages under 2 Myr are displayed in red and older elements with insertion ages over 2 Myr are displayed in blue. Black bars indicate the projected centromere positions.

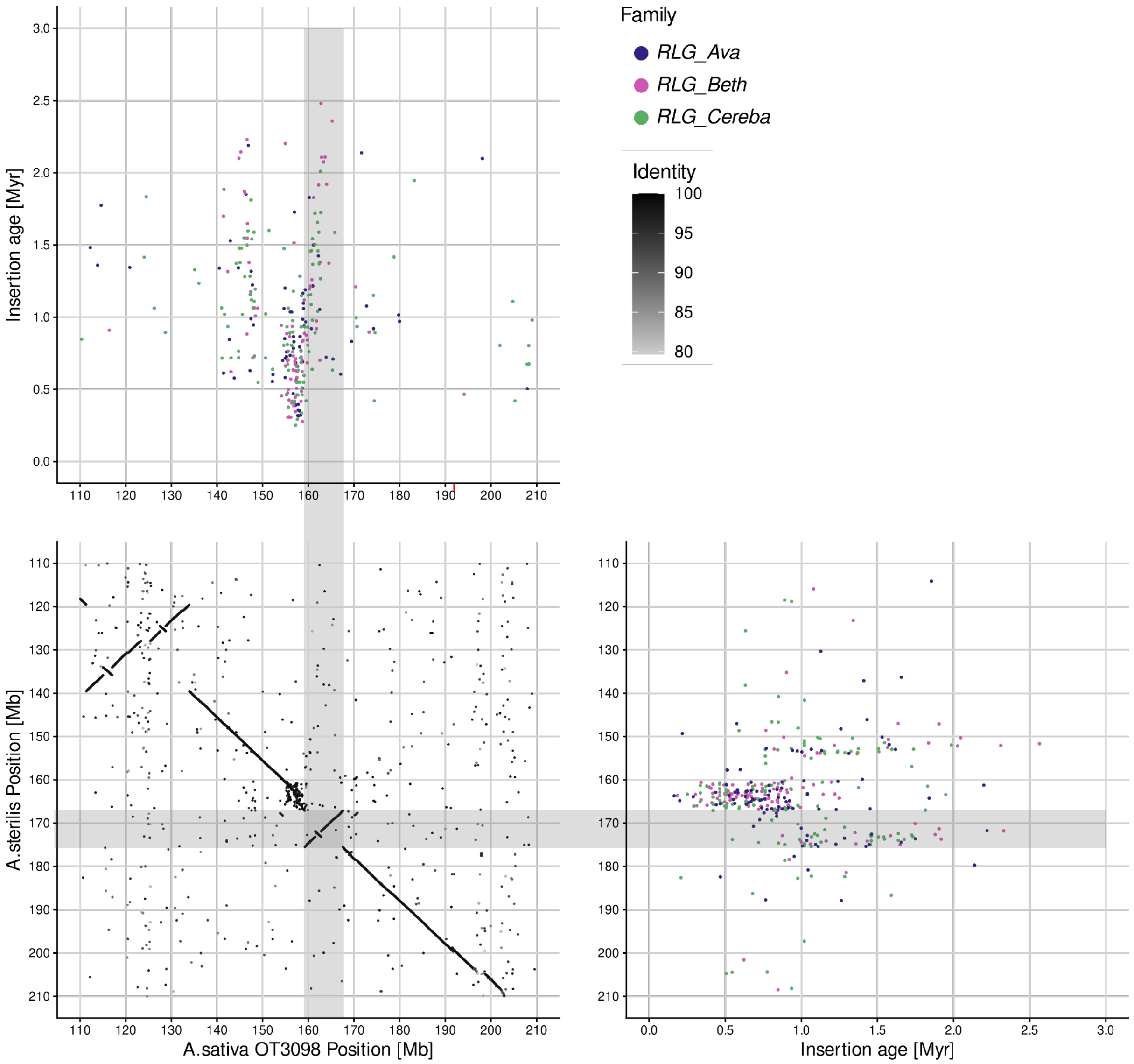

Fig. S11. Dot plot comparison of (peri-) centromeric regions of chromosomes 4D of *A. sativa* OT3098 and *A. sterilis*. Each colorful dot represents a TE insertion (*RLG_Ava* in violet, *RLG_Beth* in pink and *RLG_Cereba* in green). Sequence gaps are indicated by red marks on the y and x-axis of the insertion age distribution plots for *A. sterilis* and *A. sativa* respectively. Gray boxes indicate the approximate inversion position. Note the distinct groups of CRM TE insertions at ~ 175 Mb in *A. sterilis*.

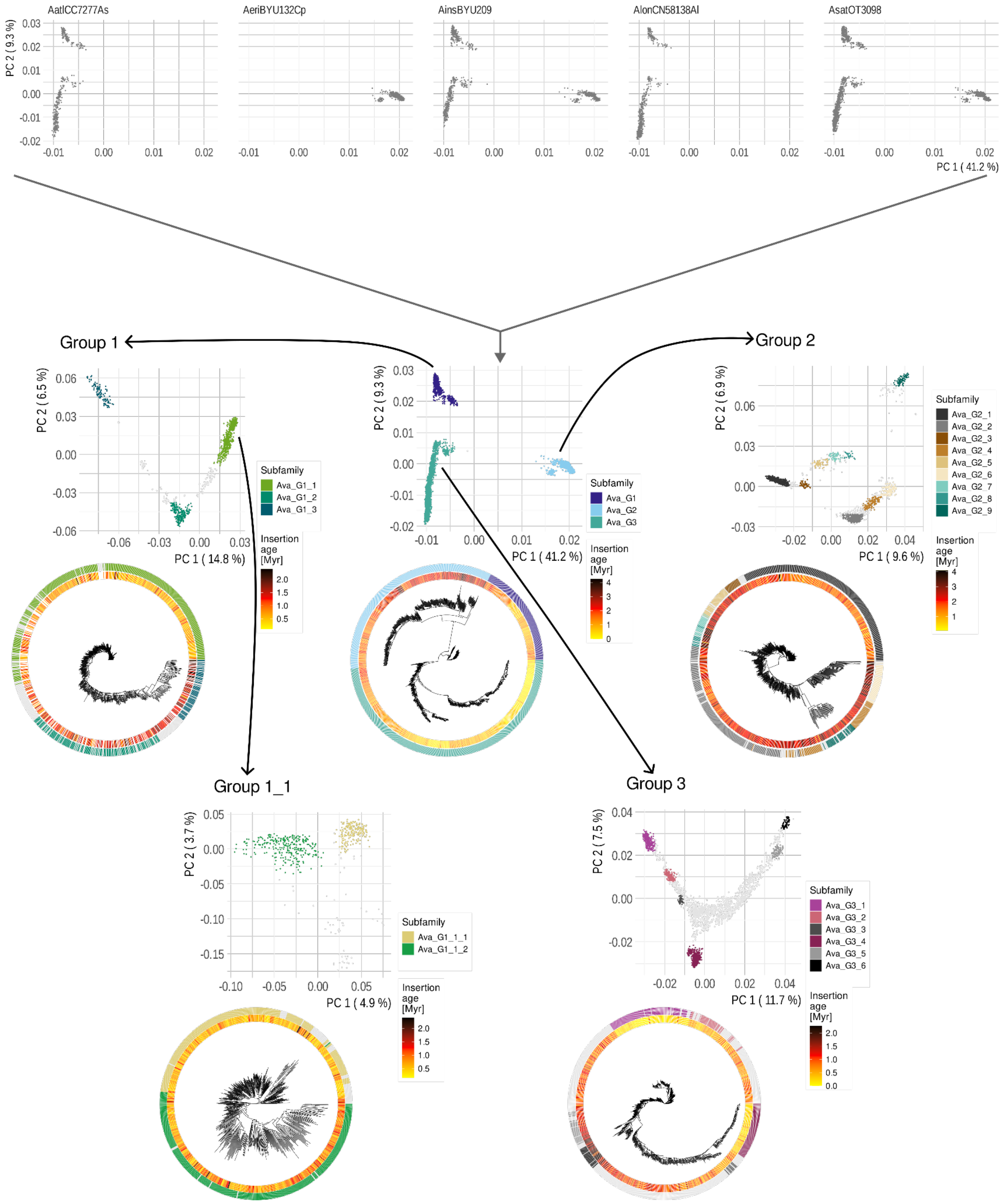

Fig. S12. PCA of *RLG_Ava* copies from *A. sativa*, *A. longiglumis* (Al), *A. atlantica* (As), *A. eriantha* (Cp) and *A. insularis* by species. The three main groups are marked in dark blue, light blue and green. Copies within these groups were used to again calculate PCAs, which enabled more detailed selection of sub-families within the scope of the main groups. The corresponding phylogenetic tree underneath each PCA was produced using RAxML with 250 randomly chosen elements per genome. If there were fewer *RLG_Ava* elements in a genome all available elements were used. Sub-families are marked in the respective color, gray indicates elements which were not sorted into a sub-family.

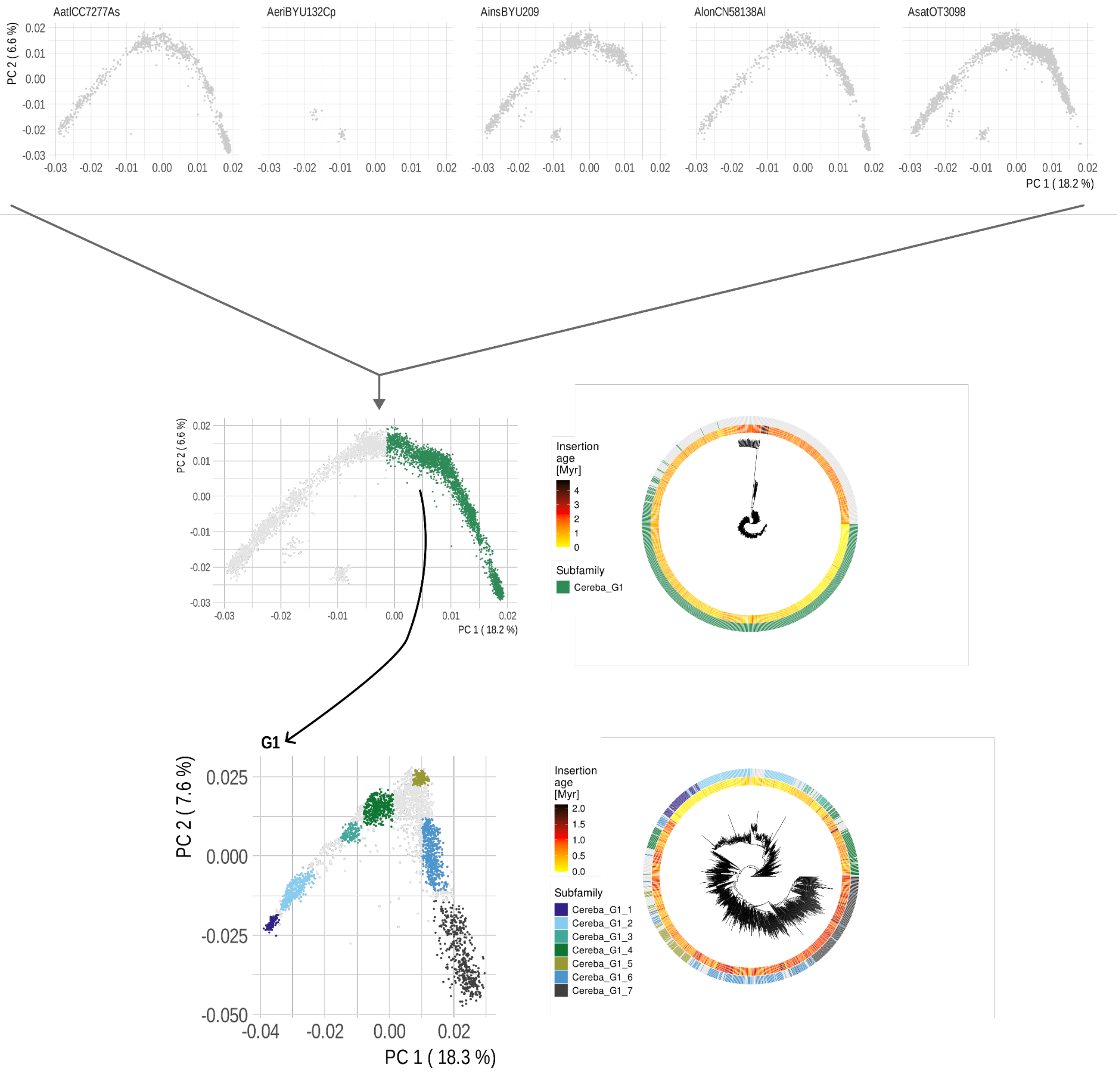

Fig. S13. PCA of *RLG_Cereba* copies from *A. sativa*, *A. longiglumis* (Al), *A. atlantica* (As), *A. eriantha* (Cp) and *A. insularis.* Elements from G1 were used to again calculate a PCA, which enabled more detailed selection of sub-families. The corresponding phylogenetic tree next to each PCA was produced using RAxML with 250 randomly chosen elements per genome. If there were fewer *RLG_Ava* elements in a genome all available elements were used. Sub-families are marked in the respective color, gray indicates elements which were not sorted into a sub-family in both, the PCA and the phylogenetic tree. PCA of all extracted full length *RLG_Cereba* copies. The elements used for further analysis are colored in their respective insertion age.

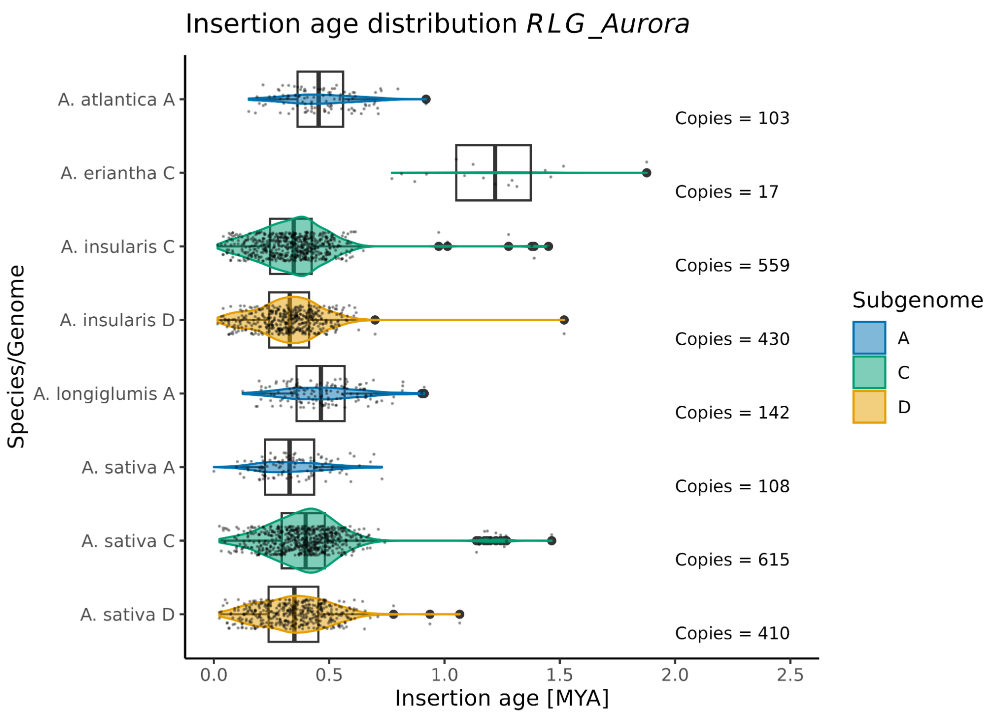

Fig. S14. Insertion age estimates for *RLG_Aurora* copies across the genomes of *A. atlantica*, *A. eriantha*, *A. insularis*, *A. longiglumis* and *A. sativa*. Copies with estimated insertion ages under 2 Myr are displayed.

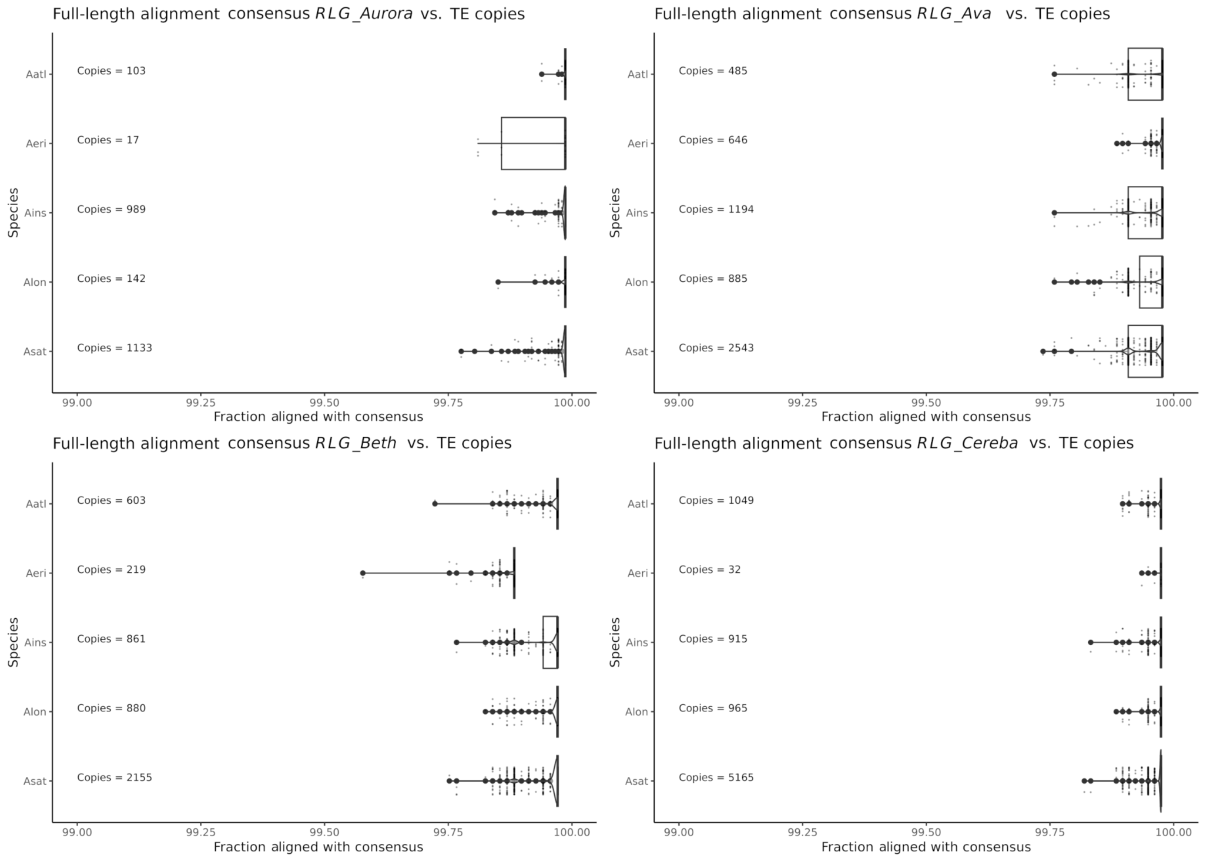

Fig. S15. Portion of consensus sequence of TE families that align with the respective TE copies. All individual TE copies of a family were aligned with the respective consensus sequence using the program Water (EMBOSS package, obtained from ubuntu.com repositories). The box plots show the proportion of the consensus sequence that aligned with each copy. In all cases, practically the entire consensus sequence can be aligned with individual copies.

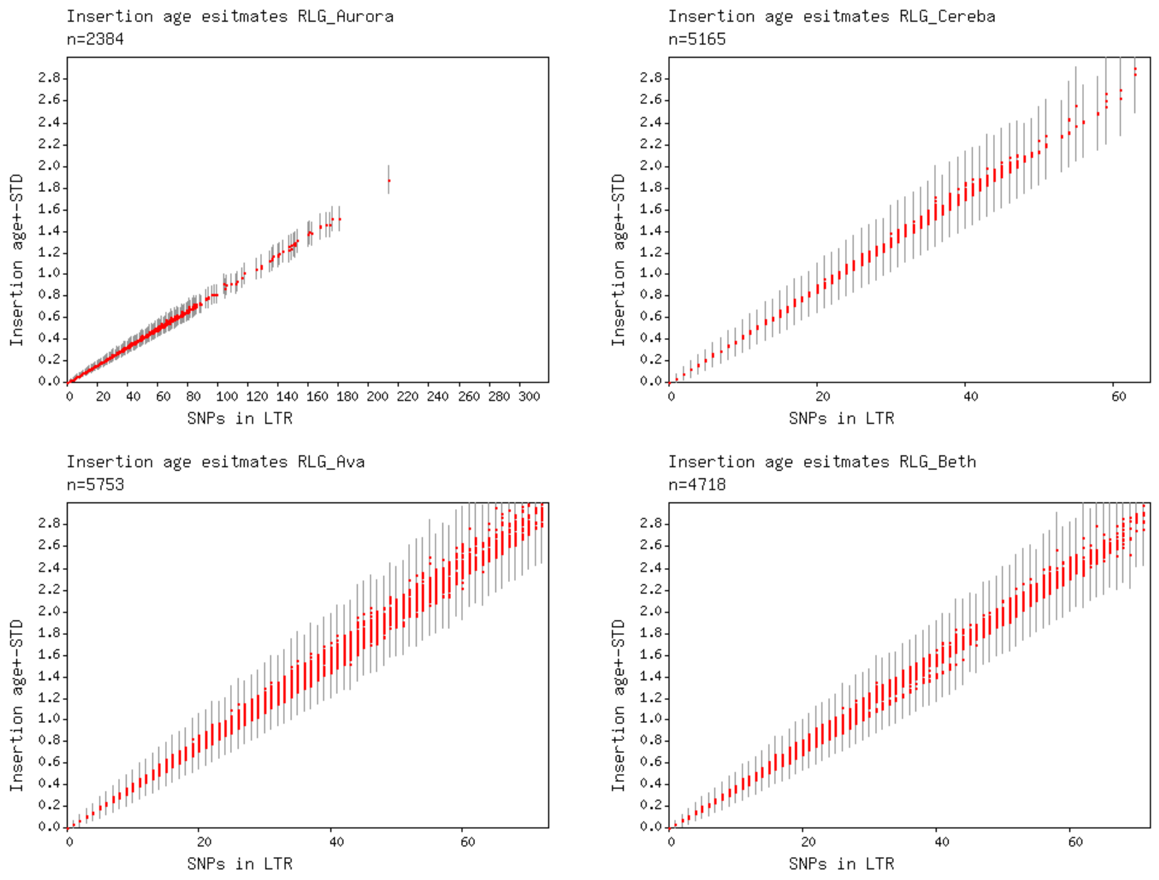

Fig. S16. Relationship between numbers of nucleotide substitutions between LTR of a full-length TE copy and inferred insertion age. The x-axis shows the total number of single nucleotide polymorphisms (transition + transversions). The y-axis shows the calculated insertion age in million years (red dots). For each insertion age estimate, the standard deviation was calculated (indicated with gray vertical lines). For longer LTRs (e.g. those for *RLG_Aurora* which are >4 kb), standard deviation consequently is smaller. Note that transition and transversions are weighted differently as previously described [54], which is why the individual insertion ages vary depending on the ratio of transition to transversions. We considered insertion age estimates older than 100’000 years as reliable since standard deviation then is in all cases smaller than the age estimate.

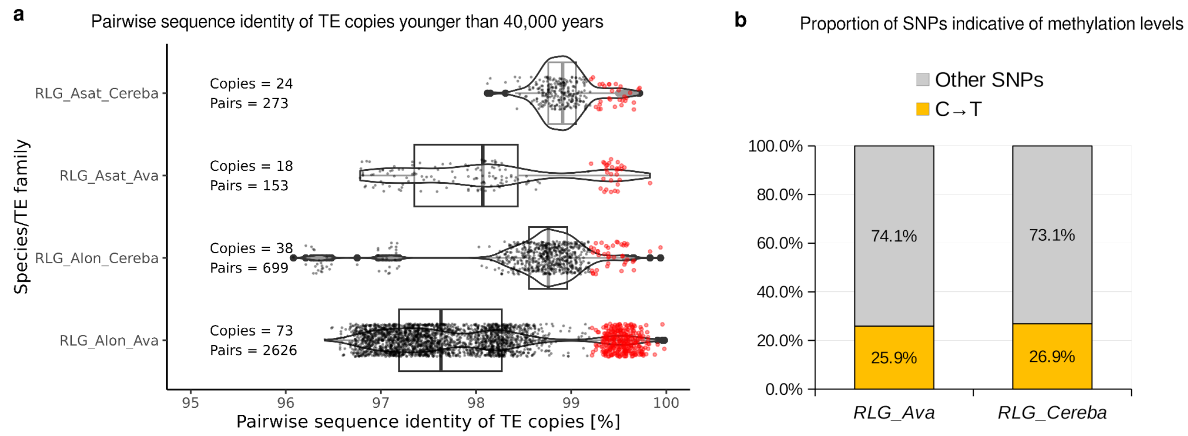

Fig. S17. Analysis of substitution rates in retrotransposon families. **a.** Within-species pairwise comparisons of TE copies. Full-length retrotransposon copies with estimated insertion ages younger than 50,000 years were aligned all vs. all across their entire length. From this, sequence identity for each pair was calculated. Highlighted in red are those pairs that show highest levels of pairwise identity and thus presumably are derived from the same active TE "master" copy. Note that sequence identity levels of these highest scoring pairs are very similar between TE families as well as between species. Boxes indicate the inter-quartile range (IQR) with the central line indicating the median and whiskers indicating the minimum and maximum without outliers, respectively. Outliers were defined as minimum – 1.5*IQR and maximum + 1.5*IQR, respectively. Asat: *A. sativa*, Alon: *A. longiglumis*. **b.** Proportions of C-to-T substitutions derived from variant calling of the young copies aligned to consensus sequences of *RLG_Ava* and *RLG_Cereba*, respectively. Shown are the percentages of C-to-T substitutions compared to all substitutions. Note that proportions are nearly the same for both families, indicating that DNA methylation levels (which typically cause higher numbers of C-to-T substitutions) are similar in both TE families.

a

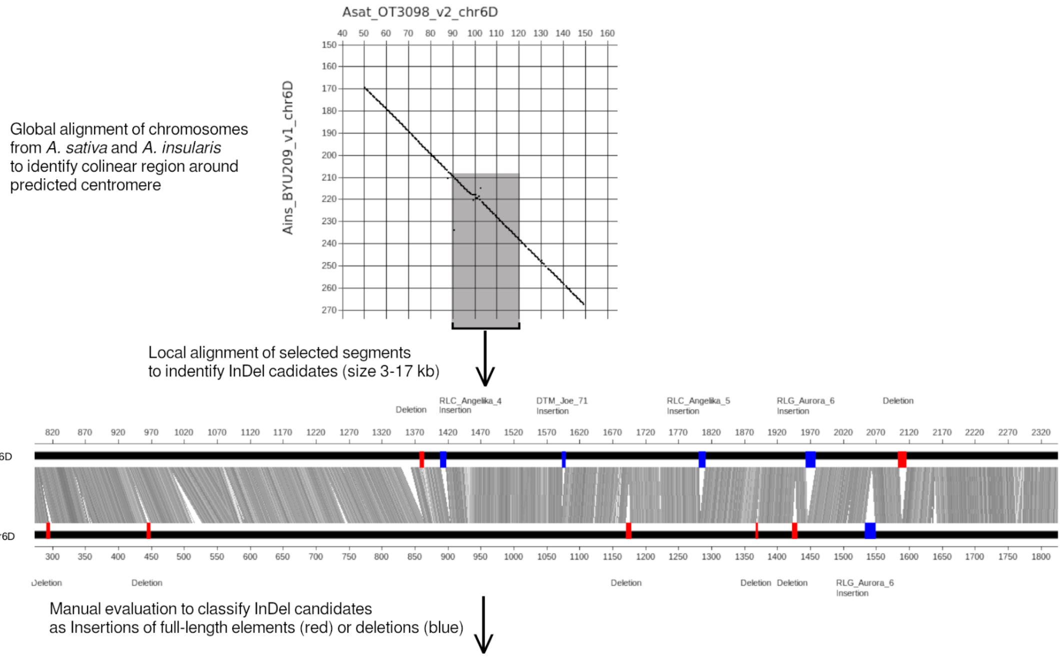

c

Chr1	Chr2	Pos1[Mb]	Pos2[Mb]	Aligned[Mb]	Insertions1	Insertions1[kb]	Deletions1	Deletions1[kb]	Insertions2	Insertions2[kb]	Deletions2	Deletions2[kb]
Asat_OT3098_v2_chr1D	Ains_BYU209_v1_chr1D	148-156	142-150	6.47	10	10390.00%	18	136.5	3	30.1	13	109.5
Asat_OT3098_v2_chr1D	Ains_BYU209_v1_chr1D	100-110	100-110	8.74	14	13610.00%	16	118.9	8	81.1	20	142.8
Asat_OT3098_v2_chr1D	Ains_BYU209_v1_chr1D	156-166	150-160	2.99	10	9480.00%	8	66	4	38.5	6	37.2
Asat_OT3098_v2_chr2D	Ains_BYU209_v1_chr2D	350-380	160-190	24.86	49	45490.00%	46	321.8	1	12.8	47	303.2
Asat_OT3098_v2_chr4D	Ains_BYU209_v1_chr4D	140-170	142-172	23.34	11	10820.00%	58	459.3	5	55	52	421.7
Asat_OT3098_v2_chr5D	Ains_BYU209_v1_chr5D	180-210	171-199	25.66	20	19730.00%	40	315.6	7	80.5	35	253.2
Asat_OT3098_v2_chr6D	Ains_BYU209_v1_chr6D	90-120	210-238	23.54	22	211	51	388.1	17	197.9	75	524.6
**Total**				**115.6**	**136**	**1306.2**	**237**	**1806.2**	**45**	**495.9**	**248**	**1792.2**

d

	Indels	Indels[kb]
Total InDel candidates	666	5400.5
Total insertions A. sativa	136	1306.2
Total insertions A. insularis	45	495.9
Total deletions A. sativa	237	1806.2
Total deletions A. insularis	248	1792.2

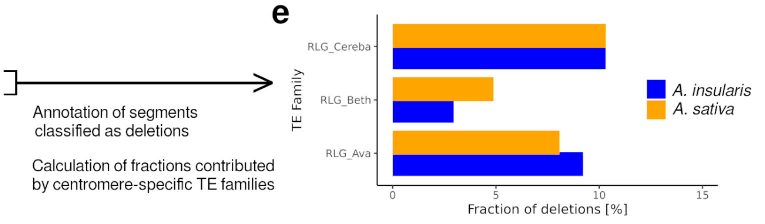

Fig. S18. Work-flow and results of the analysis of TE removal rates between *A. sativa* and *A. insularis.* a. example for global dot plot alignment of chromosome 6D in the region of the predicted centromere. From this, collinear region(s) including the centromeres were selected for subsequent local alignments. b. Example for a local alignment of a selected segment from chromosomes 6D of *A. sativa* and *A. insularis*. Sequences conserved between the two are connected by gray shaded areas. Manually curated insertion/deletions (InDels) are indicated with coloured boxes, with red boxes indicating InDels classified and deletions and blue boxes indicating those classified as insertions of full-length TEs. c. Summary data of analyzed regions with number of identified deletions and insertions in sequence 1 and 2 and their respective cumulative sizes. **d.** Summary of total numbers of InDels and their respective cumulative sizes. Note that the total numbers of deletions in *A. sativa* and *A. insularis* are very similar as well as their cumulative lengths, indicating that overall DNA removal rates are very similar in both. **e.** Contribution of the centromere-specific TE families to deleted sequences in *A. sativa* and *A. insularis.*

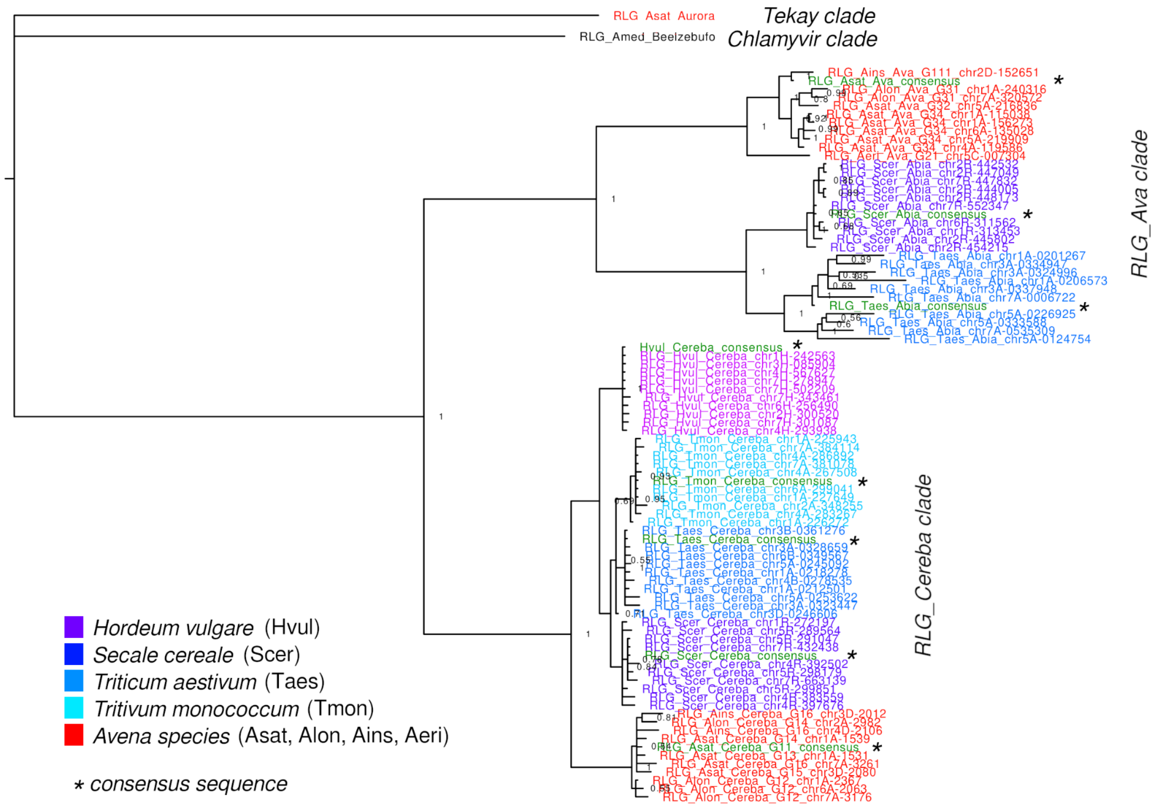

Fig. S19. Phylogenetic analysis of predicted *RLG_Ava*, *RLG_Cereba* and *RLG_Abia* integrase (INT) protein sequences from Triticeae and *Avena* species. From all species and TE families, 10 copies which encode an intact protein (i.e. no in-frame stop codons and frame shifts) were picked randomly. Additionally, the INT domain was isolated from the respective consensus sequences. The tree was produced as described in the methods. Consensus sequences are shown in green and indicated with asterisks. Note that sequences form individual TE copies cluster with their respective consensus sequences in all cases. Additionally, the phylogenetic relationship of species is reflected in both the *RLG_Ava* and *RLG_Cereba* clades. Sequences from *Gypsy* retrotransposons belonging to the *Tekay* and *Chlamyvir* clades were used as outgroups.

Supplementary Tables

Tab. S1. Centromere positions of *A. sativa* OT3098 inferred from CENH3 Chip-seq data from *A. sativa* cv. Starter.

Chr	Start	End	Chr	Start	End	Chr	Start	End
chr1A	151.58	159.69	chr1C	272.17	283.54	chr1D	143.02	151.96
chr2A	151.46	160.14	chr2C	226.43	234.8	chr2D	364.32	373.70
chr3A	233.40	243.90	chr3C	234.34	241.61	chr3D	200.73	209.67
chr4A	116.45	124.96	chr4C	359.14	368.29	chr4D	153.58	160.76
chr5A	213.56	223.83	chr5C	253.00	261.87	chr5D	186.16	194.73
chr6A	133.28	142.67	chr6C	388.68	397.53	chr6D	96.58	104.17
chr7A	320.67	330.03	chr7C	313.48	322.77	chr7D	221.73	231.67

Tab. S2. The results of comparisons of consensus sequences in *T. monococcum* and *A. sativa* OT3098

consensus in T. monococcum	consensus in A. sativa	aligned	machted	sim [%]
RLG_Tmon_Cereba_consensus-1	RLG_AsatOT3098A_Cereba_consensus-1	7237	5515	76.2
RLG_Tmon_Cereba_consensus-1	RLG_AsatOT3098C_Cereba_consensus-1	7305	5640	77.2
RLG_Tmon_Cereba_consensus-1	RLG_AsatOT3098D_Cereba_consensus-1	7255	5529	76.2
RLG_TmonTA299_Abia_consensus-1	RLG_AsatOT3098A_Ava_consensus-1	5661	3742	66.1
RLG_TmonTA299_Abia_consensus-1	RLG_AsatOT3098C_Ava_consensus-2	4720	3205	67.9
RLG_TmonTA299_Abia_consensus-1	RLG_AsatOT3098D_Ava_consensus-1	4721	3177	67.29
